# Molecular Dynamics Investigations Suggest a Non-specific Recognition Strategy of 14-3-3σ Protein by Tweezer: Implication for the Inhibition Mechanism

**DOI:** 10.3389/fchem.2019.00237

**Published:** 2019-04-17

**Authors:** Mingsong Shi, Dingguo Xu

**Affiliations:** College of Chemistry, Sichuan University, Chengdu, China

**Keywords:** molecular dynamics, protein-protein interaction, 14-3-3σ, supramolecular, tweezer, CLR01

## Abstract

The supramolecular complex formed between protein and designed molecule has become one of the most efficient ways to modify protein functions. As one of the more well-studied model systems, 14-3-3 family proteins play an important role in regulating intracellular signaling pathways via protein-protein interactions. In this work, we selected 14-3-3σ as the target protein. Molecular dynamics simulations and binding free energy calculations were applied to identify the possible binding sites and understand its recognition ability of the supramolecular inhibitor, the tweezer molecule (CLR01). On the basis of our simulation, major interactions between lysine residues and CLR01 come from the van der Waals interactions between the long alkyl chain of lysine and the cavity formed by the norbornadiene and benzene rings of the inhibitor. Apart from K214, which was found to be crystallized with this inhibitor, other lysine sites have also shown their abilities to form inclusion complexes with the inhibitor. Such non-specific recognition features of CLR01 against 14-3-3σ can be used in the modification of protein functions via supramolecular chemistry.

## Introduction

Recently, the intriguing strategies using supramolecular chemistry in the modification of protein functions via host-guest interactions have attracted extensive interests. Some prototypical applications include inhibition of proteases (Peczuh and Hamilton, 2000; D'Acquarica et al., 2011), enhancement of solution (Perret and Coleman, 2011), and manipulation of enzyme activities (Pan et al., 2018). To achieve this, surface exposed charged residues like lysine and arginine are usually used as the target recognition sites, which can form the supramolecular complexes with macrocycles, e.g., cyclo[6]aramide (Pan et al., 2018), p-sulfonatocalix[4]arene (Thomas and Ward, 2005; Perret et al., 2006), crown ethers (Shimojo et al., 2006) and cyclodextrins (Kano and Ishida, 2007). As one of typical systems, 14-3-3 proteins (Aitken, 2006) have been considered to be the perfect target used for regulation of protein-protein interactions (PPIs) via supramolecular inhibitors.

14-3-3 proteins are a unique class of regulatory, phosphoserine/threonine-binding proteins found in nearly all eukaryotic cells (Hermeking, 2006). There are seven human 14-3-3 family members (α/β, ε, η, γ, τ/θ, σ, and ζ) according to amino acid sequences (Yang et al., 2006), while plants contain up to 13 isoforms and 2 isoforms in yeast (Fu et al., 2000; Powell et al., 2003). More than one thousand binding partner proteins of the 14-3-3 domain have been identified including transcription factors, signaling molecules, tumor suppressors, biosynthetic enzymes, cell cycle regulation, cell proliferation, protein trafficking, metabolic regulation, and apoptosis (Fu et al., 2000; Aitken, 2006; Hermeking, 2006). Although they show no enzymatic activity by themselves, 14-3-3 proteins can alter the conformation, localization, stability, phosphorylation state as well as activity of a target protein via PPIs (Muslin and Xing, 2000; Tzivion et al., 2001; Aitken, 2006; Aghazadeh and Papadopoulos, 2016). Recently, 14-3-3 proteins have also been recognized to have chaperone-like functions (Sluchanko and Gusev, 2017). Binding of the 14-3-3 domain to its target proteins often occurs in a phospho-specific manner where it binds to one of two consensus sequences of their target proteins, RSXpSXP, and RXXXpSXP (Yaffe et al., 1997). On the other hand, 14-3-3 also interacts with proteins in a phosphorylation independent manner, e.g., the interaction with a specific peptide motif of the bacterial toxin exoenzyme S (ExoS) from *Pseudomonas aeruginosa* (Ottmann et al., 2007b). Their interaction is often considered to be one of the most ideal models for understanding PPIs.

The understanding of PPIs in the development of peptide-like inhibitors has been of particular pharmaceutical interest (Aeluri et al., 2014). Indeed, several attempts have been made to develop small-molecule inhibitors against the PPIs involved 14-3-3 proteins (Corradi et al., 2010; Bier et al., 2013; Thiel et al., 2013). Herein, we will discuss one of well-studied inhibitors called tweezer (**CLR01**) and how it interacts with 14-3-3σ.

The function of this inhibitor is versatile, e.g., regulating the aggregation of α-synuclein (Acharya et al., 2014), modulating the aggregation propensity and cytotoxicity of huntingtin protein (Vöpel et al., 2017), and inhibiting the activity of PARP-1(Wilch et al., 2017). The tweezer molecule is a water-soluble belt-like molecule with a torus-shaped cavity surrounded by alternating aromatic and aliphatic rings, which has the specificity of recognizing either lysine or arginine. The anionic phosphate groups on tweezer can draw the cationic side chains of lysine or arginine into its cavity to form a tight ion pair but with different binding affinity (Klärner and Schrader, 2013). The tweezer binds arginine with much weaker affinity than lysine (Fokkens et al., 2005; Dutt et al., 2013). The crystallographic structures of the 14-3-3σ/tweezer complexes were resolved by Bier et al. (2013) which shows that the alkyl chain of K214 is fully surrounded by the tweezer molecule. This can provide an ideal initial model to understand the protein-inhibitor interactions at the atomic level. Interestingly, 14-3-3σ contains 17 surface exposed lysine residues, which could also have possible stable interactions with tweezer. Indeed, the QM/MM binding energy calculation did show some other lysine residues, e.g., K27, K77, K141 and K195, can be recognized by the tweezer molecule. Meanwhile, the isothermal titration calorimetry (ITC) data also shows at least two independent binding events with different affinities when tweezer and 14-3-3σ occur together in solution (Bier et al., 2013). Obviously, only experimental ways cannot provide a full understanding of the inhibitor binding mechanism. Theoretical simulation represents one of useful ways to tackle molecular interactions from a micro-perspective. Such research has been reported in many peer-reviewed literatures on various biomolecule systems (Cao et al., 2012; Gao et al., 2015; Zhang and Zheng, 2018; Zheng and Lazo, 2018).

Herein, combining the MD simulation and binding free energy calculations, we try to locate the possible binding sites of 14-3-3σ by the tweezer molecule. This might provide a chance to understand the inhibition mechanism of protein-protein interactions from a micro-perspective, *esp*. for the 14-3-3σ protein. This could be helpful in the future development of 14-3-3σ inhibitors with better potency.

## Computational Details

### Models for Tweezer/14-3-3σ Complex

**Figure 1:**
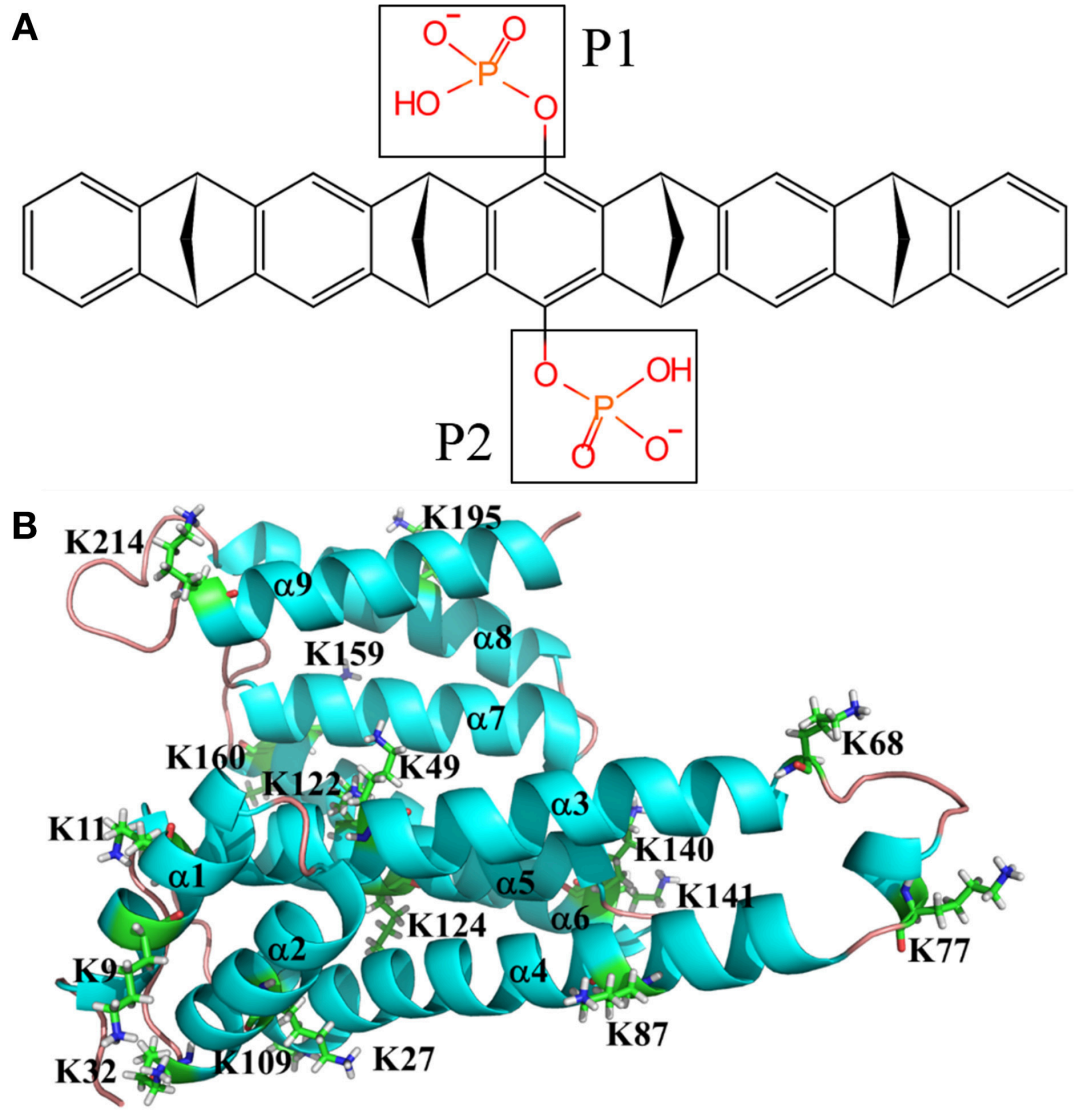
Topological depictions for 14-3-3σ monomer and tweezer molecule. **(A)** Structure of tweezer molecule. **(B)** Definitions for helices of 14-3-3σ. Cartoon style used for 14-3-3σ. The helix is defined as α1, α2, α3, α4, α5, α6, α7, α8, and α9 from N-terminal to C-terminal of 14-3-3σ (PDB entry code 5OEH).

There are many X-ray structures that have been solved for 14-3-3σ protein (Benzinger et al., 2005; Wilker et al., 2005; Molzan et al., 2010, 2013; Schumacher et al., 2010a,b; Roglin et al., 2012; Rose et al., 2012; Anders et al., 2013; Bier et al., 2013; De Vries-van Leeuwen et al., 2013; Thiel et al., 2013; Joo et al., 2015; Milroy et al., 2015; Doveston et al., 2017; Sijhesma et al., 2017; Sluchanko et al., 2017a,b; Stevers et al., 2017; Prokop et al., 2018). For example, Bier et al. (2013) reported the complex of tweezer/K214 (PDB entry code 5OEH) with a resolution of 2.35 Å. This structure will be employed in this work to simulate the interaction between the inhibitor and K214. As we have mentioned above, there are 17 surface exposed lysine residues shown in Figure 1. To fully understand the binding affinity of 14-3-3σ protein with this inhibitor, we have to analyse all possible lysine binding sites. Moreover, recent QM and QM/MM studies have shown that tweezer can form complexes with arginine or lysine residues, but that studies also displayed that the binding affinity of arginine is much weaker than lysine (Dutt et al., 2013). Therefore, only recognition status of lysine residues will be considered in this work.

In order to completely understand the interactions between all lysine residues and the inhibitor of tweezer molecule, we should consider all possible binding models. Here, an apo structure for 14-3-3σ was adopted as the initial structure [PDB code 1YZ5 (Benzinger et al., 2005) at a resolution of 2.8 Å] except K214 in the subsequent docking simulation.

The docking method was used to construct the initial complex structures. The partial charges for both receptor (14-3-3σ) and ligand (tweezer) were assigned using AutoDockTools (Sanner, 1999) with Gasteiger method (Gasteiger and Marsili, 1980). A grid map of 60 × 60 × 60 points with 0.375 Å grid spacing was generated using AutoGrid module based at the center of the α carbon atom of each lysine residues (Morris et al., 2009). The Lamarckian genetic algorithm (Morris et al., 1998) was applied as the searching approach. For each lysine sites, a total of 1,000 conformations from docking simulation were clustered according to the RMSD criterion within 2.0 Å. We carefully checked all conformations to see if the side chain of lysine can be inserted in the cavity of tweezer. The conformer with the lowest binding energy will be selected out as the initial structure for further MD simulation and binding free energy calculation. In our docking simulations, the side chain of lysine residue and tweezer were set to be flexible, whereas other protein parts remained in their crystal positions. The docking results for every lysine site can be found in the Supporting Information.

All resulted complexes were fully solvated in a rectangular box of TIP3P (Jorgensen et al., 1983) water with sodium ions added to neutralize the whole system. One typical system consists of more than 48,500 atoms with the box size about 87 Å × 74 Å × 93 Å. The periodic boundary conditions and a cut-off of 12 Å for nonbond interactions were applied. To correctly describe the long-range electrostatic interactions, the particle mesh Ewald (PME) algorithm (Darden et al., 1993) was employed. The positions of water molecules were relaxed by 9,000 steps of steepest descent and 1,000 steps of conjugate gradient minimization approach with all of the protein and ligand molecules fixed at their original positions. A further 10,000 steps of conjugate gradient full minimization were carried out for the total system. The optimized systems were gradually heated to 300 K in 200 ps in the NVT ensemble, followed by 200 ps equilibration in the NPT ensemble at 1 atm and 300 K. Subsequently, a further 300 ns MD simulations were performed for data analysis. Newton's equations of atomic motion were integrated by the Verlet algorithm with 2 fs time step. SHAKE algorithm (Ryckaert et al., 1977) was applied to constrain those covalent bonds involving hydrogen atoms. To correctly describe the tweezer molecule in our MD simulation, the standard Amber general amber force field (GAFF) (Wang et al., 2004) generation procedure was used to generate force field parameters for tweezer. First of all, the geometry of tweezer molecule was optimized at HF/6-31G level of theory using Gaussian09 suite of program (Frisch et al., 2009). The restrained electrostatic potential (RESP) protocol (Bayly et al., 1993) was employed to obtain the partial atomic charges at the B3LYP/6-31G^*^ level of theory after geometry optimization. The force field parameters for the ligands can be generated using the Antechamber program. All MD calculations were performed using the AMBER12 suite of programs (Case et al., 2012) together with AMBER ff14SB force field (Maier et al., 2015) for 14-3-3σ.

### Binding Free Energy Calculations

To quantitatively assess the binding affinity of tweezer with different lysine sites of 14-3-3σ, it would be useful to calculate the corresponding binding free energies. In this work, molecular mechanics Poisson Boltzmann (or generalized Born) surface area (MM-PB/GBSA) (Srinivasan et al., 1998; Lee et al., 2004) were employed to calculate the binding free energies.

For the calculation of binding free energy in MM-GBSA framework, it has been discussed extensively (Kollman et al., 2000; Rizzo et al., 2004; Strockbine and Rizzo, 2007; Yang et al., 2009; Hou et al., 2011). Only a short description is summarized here:

(1) $$\begin{array}{rcl} \Delta G_{binding} & = & \Delta G_{complex}-\Delta G_{14-3-3\sigma }-\Delta G_{tweezer} \end{array}$$

(2) $$\begin{array}{rcl} \text{G} & = & E_{gas}+G_{sol}-TS \end{array}$$

(3) $$\begin{array}{rcl} E_{gas} & = & E_{int}+E_{vdW}+E_{ele} \end{array}$$

(4) $$\begin{array}{rcl} G_{sol} & = & G_{GB}+G_{np} \end{array}$$

Δ*G*_*complex*_, Δ*G*_14−3−3σ_ and Δ*G*_*tweezer*_ are denoted as free energies of the complex (tweezer/K), the protein (14-3-3σ) and the ligand (tweezer), respectively. For MM-GBSA approach, only one MD trajectory is needed to get these values via a statistical average way. The solvation free energy (*G*_*sol*_) can be principally divided into both polar (*G*_*GB*_) and non-polar (*G*_*np*_) terms. The non-polar solvation free energy comes from the surface formation and van der Waals interactions between the solute and solvent, which can be evaluated by the equation of γ*SA* + *b*, where γ = 0.0072 kcal·Å^−2^ and *b* = 0.0 kcal·mol^−1^. The *SA* denotes the solvent's accessible surface area, which was estimated using the program MSMS (Sanner et al., 1996). The polar solvation part is calculated by solving the Generalized Born equation (Onufriev et al., 2000). Here, dielectric constants for the solute and solvent were set as 1 and 80, respectively. For each complex system, binding energies were averaged over 2,000 frames of the last 200 ns MD trajectory. Entropy can be estimated from changes in the degrees of freedom including translation, rotation, and vibration. These entropy terms are functions of the mass and moments of the inertia of molecule, thus can be easily calculated using the standard equations of statistical mechanics. Specifically, contributions to the vibrational entropy were calculated using the normal mode analysis approach (Case, 1994). The −*TS* was averaged over 200 snapshots of the MD trajectory.

## Results

As we have mentioned above, there are total of 17 lysine residues located on the surface of 14-3-3σ. A thorough investigation of recognition ability of the tweezer against all these lysine sites could provide a complete understanding of the interactions between the tweezer molecule and the protein. To get an accurate binding affinity comparison, a 1:1 inclusion ratio was employed in this work. Moreover, except tweezer/K214 complex model, all other initial complex models were prepared by a docking approach using AutoDock tool. All initial models (Figures S1-S11 and S13-S16) after AutoDock can be found in supplementary file. Subsequent 300 ns separate MD simulations were carried out to get fully relaxed complex structures.

**Figure 2:**
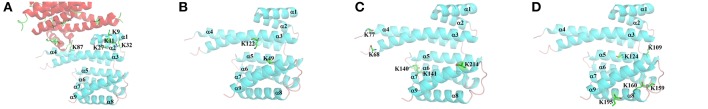
Cartoon representation for the classification of all surface exposed lysine residues of 14-3-3σ. Two chains are colored in red and cyan, respectively. **(A)** I-type, **(B)** II-type, **(C)** III-type, and **(D)** IV-type.

According to the dimeric characteristics of the 14-3-3σ protein, we simply classify these lysine residues into four classes for convenience. The first class (I-type) contains the lysine residues existing at the 14-3-3 dimer interfacial region, which includes five lysine residues like K9, K11, K27, K32, and K87. The second class (II-type) consists of lysine residues (K49 and K122) located inside the 14-3-3σ active cavity. The third class of lysine residues (III-type) is located at the edge of binding groove. Five lysine residues, K68, K77, K140, K141 and K214, belong to this class. The fourth type of lysine residues (IV-type) locates on the reverse side of the 14-3-3σ protein active groove, which includes K109, K124, K159, K160, and K195. To illuminate our classification, a schematic plot to show all lysine residues was given in Figure 2.

### Binding With K214

**Figure 3:**
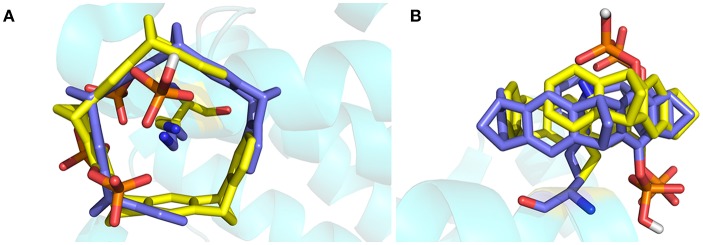
The superimposed structures of the X-ray structure and the docked structure for tweezer included K214. The tweezer molecule and K214 are plotted using stick style, in which carbon atom is colored in yellow for X-ray tweezer, while blue for the structure after docking. **(A)** Top view of the superimposed structures, **(B)** side view of the structures.

The crystal structure of 14-3-3σ complexed with tweezer has been reported. Therefore, it would be better to compare both docking model and the X-ray structure first. As shown in Figure 3, we can find out that except the orientation, the tweezer molecule in two models locates nearly at the same position, and can recognize the lysine residue very well. One of the phosphonate groups is coordinated by the lysine residues as expected. Due to its belt-like or torus-shaped topology of tweezer molecule, possible rotation around the side chain of K214 could happen when it binds lysine residues. Therefore, we believe all docked models obtained using AutoDock 4.2 (Morris et al., 2009) can be applied in our dynamic simulation to tackle the recognition mechanism. Of course, for the tweezer/K214 complex model, the X-ray structure [PDB entry code 5OEH (Bier et al., 2013)] will still be employed as the initial model.

**Figure 4:**
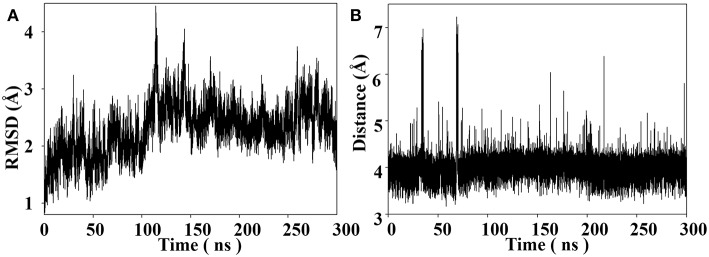
**(A)** Backbone RMSD as a function of simulation time for the tweezer/K214 complex system with the X-ray structure as the reference. **(B)** Distance between the nitrogen atom of amine group of K214 and the phosphorus atom of the phosphate group of the tweezer molecule throughout the MD simulation.

**Figure 5:**
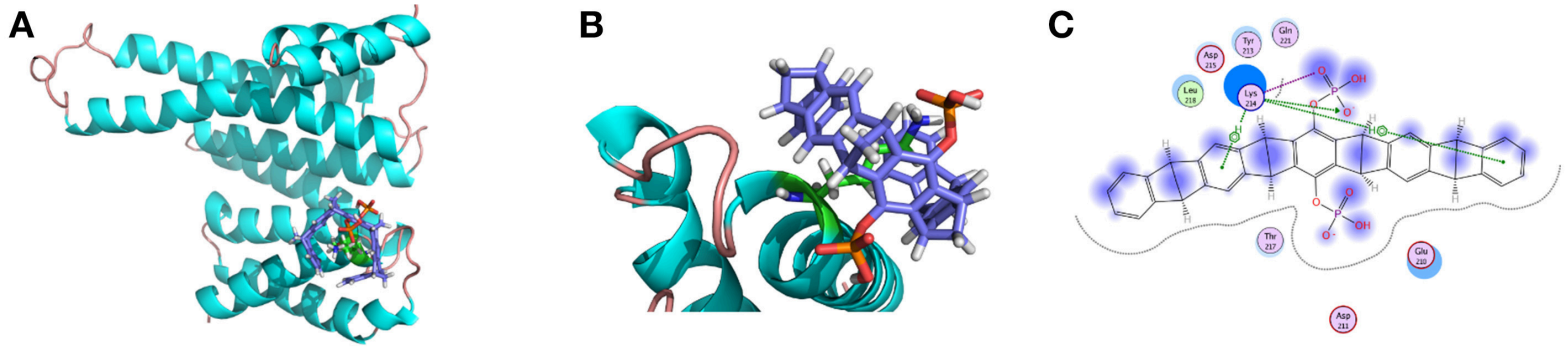
**(A)** Snapshot of tweezer/K214 complex extracted from the MD trajectory of the centroid conformation of the maximum cluster of the last 200 ns. **(B)** Zoom-in view representation. **(C)** 2-D Protein-ligand interaction depictions of K214/tweezer complex by the end of MD simulation (300 ns). Green circles represent hydrophobic residues, purple circles represent polar residues, red ring represent acidic residues, and blue interior rings represent basic residues. Hydrogen bonding interactions are drawn with an arrowhead, sidechain in green and backbone in blue. Green benzol rings with a “+” describe an arene–cation binding; two benzol rings, an arene–arene binding. The solvent accessible surface area is plotted onto the atoms in blue smudge for ligand and over the constituent atoms as turquoise halo for receptor residues, respectively. (For detailed interpretation of the color definitions, the reader is referred to the web version of this article (Clark and Labute, 2007).

Throughout the 300 ns MD simulation, the overall 14-3-3σ protein maintains its topology very well, judged by the backbone atom root mean square deviation (RMSD) of 2.30 ± 0.44 Å plotted in Figure 4A. One snapshot extracted from the MD trajectory was depicted in Figures 5A,B. As shown in Figure 5A, K214 is situated at α9, which stays at the edge of the protein binding groove. Once the tweezer/K214 inclusion complex is formed, there is no big fluctuation for the overall conformation of the protein during the dynamics. The side chain group of K214 can stay inside the cavity of tweezer molecule very stably via various interactions. First of all, a stable ion pair is formed between one of negatively charged phosphate groups (P1) of tweezer and the positively charged amino group of lysine residue, judged by the distance of *d*_*P*−*N*_ = 4.02 ± 0.28 Å in Figure 4B. Although occasionally flexibility of this phosphate group can be observed during the simulation, further dynamics running can recover their interaction. Except the positively charged amino group of lysine residue, its long alkyl chain forms quite stable hydrophobic interactions with both norbornadiene and benzene rings. In fact, the topological structure of tweezer requires the recognized residue to have a long side chain. Clearly, lysine and arginine are two residues that match the requirement. Interestingly, some other supramolecules like calixarene (Perret et al., 2006) or cyclo-[6]aramide (Pan et al., 2018) shows their recognition specificity with lysine or arginine in cytochrome c.

On the other hand, a relatively weak hydrogen bond network formed between the second phosphate group (P2) and 14-3-3σ can be observed, judged by the hydrogen bond occupancy analysis, e.g., only 22% of dynamic time between the hydroxy of second phosphate group and the carboxylate of D215. In fact, this phosphate group is exposed to water during most of the dynamic time. Furthermore, to shed more light onto the interaction patterns between the tweezer molecule and surrounding residues, a diagrammatic plot was given in Figure 5C. Besides the salt bridge formed between K214 and the phosphate group (P1) of tweezer molecule, major interactions should be attributed to the long alkyl chain of K214 with the benzene and norbornadiene units of tweezer. Moreover, the tweezer/K214 complex seems to be surrounded by other residues like E210, Y213, D215, T217 and L218, although no direct interactions like hydrogen bond can be observed.

Of course, MD simulation can only provide qualitative descriptions of the substrate binding information. Further quantitative assessment of the recognition ability of 14-3-3σ by tweezer is highly necessary. Here, the binding free energy was obtained by MM-GBSA method. As listed in Table 1, $\Delta G_{bind\;}^{calc}=$ −2.54 kcal·mol^−1^ was obtained for tweezer/K214 complex, which has as large as ~4–6 kcal·mol^−1^ difference from experimental data. Such big difference from experimental observation might suggest that K214 is not the preferred binding site by tweezer molecule in solution phase. In fact, such an observation has also been realized by Bier et al. (2013). These authors carried out classical MD simulations for the tweezer/K complexes, and then calculated their total energies via B3LYP-D2/CHARMM method, which were then used to rank the binding possibility for different lysine sites. They further proposed some other lysine binding sites even better than K214 could also be reached by the tweezer molecule. Our results based on MM-GBSA binding free energy calculation can reproduce their conclusion that there is not only one lysine site for tweezer. In other words, more comprehensive dynamics simulations and corresponding binding affinity investigations should be carried out for other lysines of 14-3-3σ protein.

**Table 1:** Binding free energy for tweezer/K214 complex and decomposition to electrostatic interaction, van der Walls interaction, solvation free energies, and entropy.

	**Complex**	**Receptor**	**Ligand**	**Delta**
*E*_*vdW*_	−1030.74 (19.99)	−1000.56 (19.61)	3.62 (1.17)	−33.80 (3.09)
*E*_*ele*_	−6047.66 (151.07)	−6094.35 (136.52)	−205.99 (3.60)	252.69 (29.08)
*E*_*GB*_	−4988.24 (130.87)	−4566.88 (118.60)	−181.83 (2.04)	−239.53 (25.73)
*E*_*surf*_	96.48 (1.85)	95.60 (1.82)	4.68 (0.07)	−3.79 (0.25)
*G*_*gas*_	−1298.46 (147.82)	−1617.38 (134.66)	100.06 (6.95)	218.85 (28.42)
*G*_*solv*_	−4891.76 (130.45)	−4471.28 (118.05)	−177.15 (2.04)	−243.33 (25.82)
*E*_*gas*_+*G*_*sol*_	−6190.22 (47.05)	−6088.66 (46.19)	−77.09 (6.78)	−24.47 (4.13)
*TS*_*total*_	2628.30 (7.61)	2578.96 (7.37)	71.26 (0.12)	−21.93 (2.25)
$\Delta G_{bind}^{cal}$				−2.54
$\Delta G_{bind}^{\exp}$				−8.9 ~-6.2

*Energies are in kcal·mol^−1^. Numbers in parentheses are standard deviations*.

Nevertheless, we still have some useful information based on MD results for tweezer/K214 complex. Basically, the binding free energy is generally decomposed to polar (Δ*E*_*ele*_ + Δ*E*_*GB*_) and non-polar (Δ*E*_*vdW*_ + Δ*E*_*surf*_) contributions. According to Table 1, total polar contribution is calculated to be 13.16 kcal·mol^−1^, while −37.59 kcal·mol^−1^ for non-polar contribution. Although an ion-pair is formed between K214 and tweezer, the overall electrostatic interaction between protein and tweezer shows a large positive number. This cannot be mediated by solvent effect. Therefore, we might simply consider that the ion pair can only function upon positioning the tweezer molecule. Such an observation also agrees with our hydrogen occupancy analysis. On the other hand, the van der Waals interaction has a significant stabilization effect during the inclusion complex formation. At the same time, considering the absolute values of Δ*H* and *TΔS*, the inclusion of 14-3-3σ by tweezer is an enthalpy driven pathway.

We have mentioned above that some residues can be found to be very important during the binding of tweezer according to 2-D Protein-ligand interaction depictions shown in Figure 5C. To get a more quantitative understanding of contributions from all these residues, we further conducted the binding free energy decomposition calculations per residue. Corresponding results can be found in Table S1. As large as −8.98 kcal/mol can be found for the contribution from K214, which means K214 constitutes the major binding free energy. However, D214 shows 2.31 kcal/mol to the binding, which is completely negative to whole substrate binding. Other residues like E210, T217, and L218 show some moderate contributions to binding affinity. Obviously, the relatively low binding affinity found for K214 by tweezer based on our simulation could be a combination effects from all residues.

### Binding With I-type Lysines

**Figure 6:**
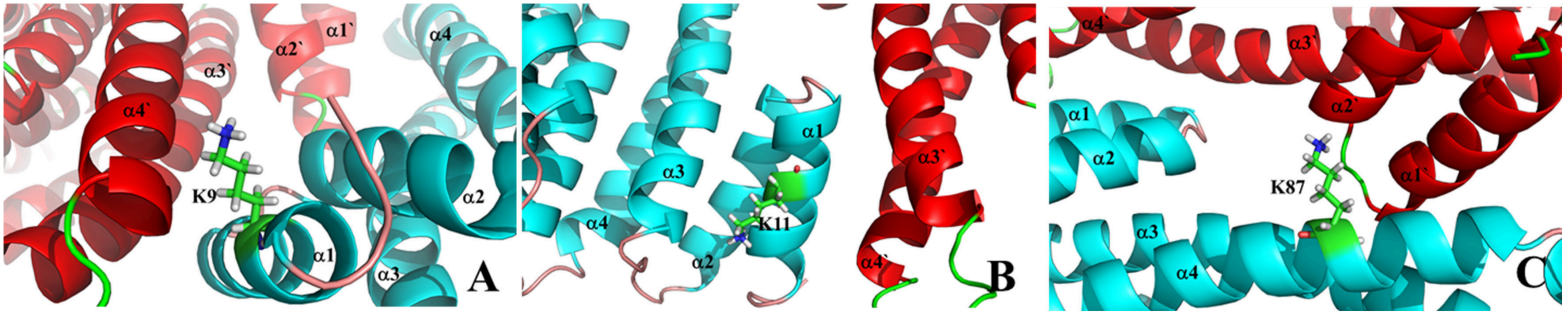
The zoom-in view for I-type lysine residues on the dimer interface of 14-3-3σ dimer. **(A)** K9, **(B)** K11 and **(C)** K87.

It is well known that the active 14-3-3 protein should be in its dimer structure (Jones et al., 1995; Messaritou et al., 2010; Woodcock et al., 2015). However, previous study has shown that the inhibitors can be bound around the dimer interfacial region and thus disrupt the function of 14-3-3 proteins (Ehlers et al., 2018). Therefore, it would be useful to carry out MD simulation to understand the binding characteristics for those lysine residues at the interface between two monomers. Here, we only employ the monomer structure in our simulation, which could be used to evaluate the binding affinity compared to other lysine sites. The **I**-type includes the lysine residues such as K9, K11, K27, K32, and K87, which distribute on several helices (α1~α4). As shown in Figure 6, K9 and K11 locate at the middle of α1, and are also deeply embedded into two helices of two protein chains. On the other hand, the side chain of K87 points toward the cavity formed between two monomer chains. We can simply postulate that these three lysine residues could not be easily reached by the tweezer molecule on the basis of its size. To this point, we then focus on the K27 and K32 for the possible **I**-type lysine binding sites. Corresponding snapshots extracted from MD trajectories are plotted in Figure S17.

According to the original crystal structure, the side chain of K27 seems impossible to be recognized by tweezer molecule. However, due to flexible docking strategy, we can construct a stable initial model of tweezer/K27 inclusion complex. Indeed, thanks to the strong interaction between alkyl chain of lysine and norbornadiene/benzene units of tweezer, the side chain of K27 stays inside the cavity of tweezer molecule quite stably throughout the MD simulation. On the other hand, a stable ion pair can still be formed between the phosphate group and the amino group of K27, evidenced by the distance of *d*_*P*−*N*_ = 4.05 ± 0.15 Å in Table 2. Besides this, the hydrogen bond formed between one of the two phosphate groups of the tweezer molecule and imidazole group of H106 can be observed during the simulation. A total of 42.87% hydrogen occupancy rate was found for the interaction between H106 and tweezer. To get a quantitative assessment for the binding affinity, we have also calculated the binding free energy using MM-GBSA method. As listed in Table 2, the binding free energy is calculated to be $\Delta G_{bind}^{calc}$ = −12.78 kcal·mol^−1^ for K27 recognized by tweezer, which means much stronger binding than K214. Similar to the recognition at the site of K214, the non-polar terms play the major contribution. According to Table S2, the polar (Δ*E*_*ele*_ + Δ*E*_*GB*_) and non-polar (Δ*E*_*vdW*_ + Δ*E*_*surf*_) contributions were calculated to 7.47 kcal·mol^−1^ and −43.53 kcal·mol^−1^, respectively.

**Table 2:** Binding free energies for tweezer/K complexes and the ion pair distance.

**Lysine**	**Type**	$\Delta {\text{G}}_{\text{b}in\text{d}}^{\text{c}al\text{c}}$	**d_P−N_**
9	I	N.A	N.A
11	I	N.A	N.A
27	I	−12.78	4.05 ± 0.15
32	I	−9.29	3.97 ± 0.20
49	II	N.A	N.A
68	III	−4.84	4.58 ± 0.99
77	III	−12.84	4.22 ± 0.77
87	I	N.A	N.A
109	IV	−6.62	4.00 ± 0.18
122	II	N.A	N.A
124	IV	N.A	N.A
140	III	N.A	N.A
141	III	−12.80	4.01 ± 0.18
159	IV	−9.26	4.29 ± 0.73
160	IV	−3.84	3.94 ± 0.20
195	IV	−14.31	4.03 ± 0.17
214	III	−2.54	4.50 ± 0.91
Exp.		−8.9 ~-6.2	

Energies are in kcal·mol^−1^ and distance in Å.^*^

^*^ΔG is the binding free energy calculated by MM/GBSA method.

D_N−P_ denotes the distance between the phosphate group of tweezer and the amino group of lysine residue.

On the other hand, the K32 is located at the terminal region of α2. Its side chain could show relatively large flexibility, which makes the K32 easily recognized by tweezer during MD simulation. As we can see from Figure S17, the alkyl side chain of K32 is stably inserted into the cavity of tweezer molecule. At the same time, the stable ion pair is maintained between the negatively charged phosphate group and the positively charged amino group with *d*_*P*−*N*_ = 3.97 ± 0.20 Å. A large binding free energy for tweezer/K32 ($\Delta G_{bind}^{calc}$ = −9.29 kcal·mol^−1^) also indicates that K32 cannot be ignored for the inhibitor design.

Although K27 and K32 are located at the interface between two monomer chains, their positions still allow them to be recognized by tweezer. Moreover, the binding free energy for these two lysine residues indicates much stronger binding affinity than the tweezer/K214 complex. On the basis of our simulation, those residues located around the interfacial region of two proteins cannot be simply ignored. Such kinds of observations are not unique in 14-3-3 proteins. Protein engineering trials have been proposed for residues located at the dimer interface for 14-3-3 proteins, since the dimeric structure was proposed to be essential for the activity and functions of 14-3-3 proteins (Messaritou et al., 2010). For example, a previous site directed mutagenesis study showed that the dimeric status of 14-3-3ζ proteins is regulated by the phosphorylation of S58, which is situated within the dimer interface (Woodcock et al., 2003). Recently, the ligand that targets the dimerization interface of the 14-3-3 adapter protein has been reported by Ehlers et al. (2018). Moreover, the disruption of dimeric 14-3-3 structure by the sphingosine-like molecules and N-alkylated trimethyl ammonium molecules has been proposed to be a novel method to the anti-cancer therapeutics (Woodcock et al., 2015).

### Binding With II-type Lysines

Two lysine residues including K49 and K122 belong to **II**-type. The K49 plays an important role in the binding between 14-3-3 proteins and the binding partner proteins from the analyses of nine crystal structures (Thiel et al., 2013). At the same time, if the active site of 14-3-3σ, which includes the K49 and K122, is occupied and competitively blocked by the small molecules, the activity of the partners will be changed accordingly (Würtele et al., 2003; Zhao et al., 2011).

As displayed in Figure 2B, K49, and K122 stay deeply inside the active site of the 14-3-3σ protein, which shows a deep internal amphipathic groove formed by several helices like α3, α5, α7, and α9. In particular, K122 locates on α5, which forms the bottom of the binding groove of 14-3-3σ protein together with α7. On the other hand, the helices of α3 and α9 constitute two side faces of the binding groove, which result that there is not enough space to accommodate the tweezer molecule in Figure S9. Therefore, the recognition of K122 by the tweezer molecule should not be expected, and no further simulation will be conducted here. K49, on the other hand, is found to be in the middle of α3, which can be encapsulated by the tweezer cavity after the initial AutoDock simulation in Figure S3. However, this inclusion complex shows instability during the MD simulation. According to the geometry evolution along the MD time, we can find that side chain of K49 does not stay inside the tweezer cavity anymore. To illustrate this, we then plotted five snapshots along the dynamics time course in Figure S4. Clearly, the tweezer molecule cannot form a stable inclusion complex with either the K49 or K122. Therefore, we might simply discard further discussion of **II**-type lysine binding sites.

### Binding With III-type Lysines

Fundamentally, the binding groove of 14-3-3σ is a canyon-like shape. All lysine residues belonging to **III**-type, K68, K77, K140, K141 and K214, can be found at the edge of the groove from Figure 2C. Since the tweezer/K214 complex has been discussed to some extent above, we will focus on the rest of the lysines in this section. Their snapshots obtained from MD trajectories are illustrated in Figure S18.

K68 locates in the loop region between α3 and α4. Throughout the MD simulation, the side chain of K68 can be included by the cavity of tweezer, but shows relatively large flexibility. Indeed, much larger fluctuation and longer average distance can be found for the ion pair (*d*_*P*−*N*_ = 4.58 ± 0.99 Å), which resulted in a weak recognition ($\Delta G_{bind}^{calc}$ = −4.84 kcal·mol^−1^) in Table 2. Nevertheless, as shown by the 2-D protein-ligand interaction diagram in Figure S6, a similar interaction pattern between K68 and tweezer can be still observed compared to tweezer/K214 complex.

We can also locate the same interaction patterns in other type-**III** lysine sites, although different recognition ability can be found. For example, the K77, which belongs to the type-**III** lysines, is located at the same loop region as K68. During the MD simulation, the side chain of K77 is deeply embedded into the cavity of tweezer molecule, but forms a very strong ion pair via its amine group and the phosphate group with *d*_*P*−*N*_ = 4.22 ± 0.77 Å. On the other hand, it shows much stronger recognition ability than K68. The calculated binding free energy for tweezer/K77 complex is about −12.84 kcal·mol^−1^. The ion pair distance for the tweezer/K68 complex is about 0.36 Å longer than that in the tweezer/K77 system. A common residue of E76 seems to cause the difference. From Tables S7, S9, we can see that the E76 contributes negatively to the stability of the tweezer/K68 system, while positively to the tweezer/K77. Structurally, the E76 shows no direct contact with two lysines or the tweezer molecule. Therefore, the different contributions of E76 to the binding free energies might come from its interactions with other protein residues.

The other two type-**III** lysine residues, which are located at the edge region of active groove, are K140 and K141. However, according to our MD simulation, the side chain of K140 cannot be well enclosed by the tweezer cavity in Figure S12. On the contrary, K141 can form a quite stable complex with tweezer judged by $\Delta G_{bind}^{calc}$ = −12.80 kcal·mol^−1^. Again, the interaction patterns between K141 and the tweezer are much like the binding of tweezer/K214.

Those lysine residues located the edge of the amphipathic groove of 14-3-3σ can form inclusion complexes with the tweezer so that the space of groove will be changed to affect the activity of the chaperone protein 14-3-3σ. Thus, the partner proteins are inactivated either by cofactor capture or by complexation with lysine residues, most likely in the vicinity of the active site, which shows for the III-type lysine residues (Wilch et al., 2017). According to the crystal structure of 14-3-3/ExoS (Ottmann et al., 2007b; Karlberg et al., 2018) and tweezer/K214 (Bier et al., 2013), we can find that the C-terminal of the ExoS is occupied at the same position for tweezer, which further indicates that the 14-3-3/ExoS PPI system could be disrupted by the tweezer molecule and result in the loss of activity of ExoS.

### Binding With IV-type Lysines

In addition to the above lysine residues, other lysine residues are located at the reverse side of the active groove of 14-3-3σ. Further, those lysine residues are also far away from the dimer interface of 14-3-3σ. Therefore, their recognition status cannot have direct impact to the PPIs involved 14-3-3σ. However, if these lysine residues can be recognized by tweezer, substantial conformational change might be expected.

First of all, the K124 is found to occur in the α5, which cannot be well recognized by the inhibitor due to obvious steric hindrance as shown in Figure S10. For other **IV**-type lysine residues like K109, K159, K160 and K195, quite stable inclusion complexes formed with tweezer can be obtained via our long time MD simulations. Figure S19 plots all snapshots for the IV-type lysines complexed with tweezer molecule. Not surprisingly, the interaction patterns with tweezer are generally the same. For example, stable ion pairs are formed between one of phosphate groups of tweezer molecule and the –${\text{NH}}_{3}^{+}$ group of lysine, evidenced by *d*_*P*−*N*_ = 4.00 ± 0.18 Å for K109, 4.28 ± 0.73 Å for K159, 3.94 ± 0.20 Å for K160 and 4.03 ± 0.17 Å for K195. The long alkyl chain of lysine can be well accommodated by the cavity formed by benzene/norbornadiene rings. Binding free energy calculations also confirm that these residues can be bound within the cavity of the tweezer molecule, albeit the binding affinities have some differences, e.g., −6.62 kcal·mol^−1^ for K109, −9.26 kcal·mol^−1^ for K159, −3.84 kcal·mol^−1^ for K160 and −14.31 kcal·mol^−1^ for K195. Notably, **IV**-type are not close to the conventional 14-3-3 protein binding groove. Such pretty strong binding affinities might indicate that the recognition of the lysine residues by the tweezer might not have specificity, if we take all above binding free energy calculations together.

## Discussion

As we have presented above, the recognition patterns of surface exposed lysine residues of 14-3-3σ by the tweezer (CLR01) molecule were explored using MD simulation approach. Our results clearly suggest that pretty stable supramolecular complexes can be formed between 14-3-3σ and the inhibitor. First of all, due to hydrophobic characteristics of the inner concave cavity formed by benzene and norbornadiene rings of the ligand, the long alkyl side chain of lysine seems to be the major factor to provide the binding affinity. Apart from this, additional stabilization comes from the stable ion pair formed between the phosphate group of CLR01 molecule and the amine group of lysine residue. In particular, the MD simulations and binding free energy calculations also indicate that this small molecule can recognize 10 of 17 lysine residues very well, which means no preferential lysine recognition site, although the reported X-ray structure shows co-crystallization of only K214 with the inhibitor. This is not unexpected, since previous QM and QM/MM MD simulations have also suggested that there has to be the second lysine-binding site for this tweezer molecule. At the same time, the isothermal titration calorimetry (ITC) measurements clearly revealed a two-phase course, which means at least two independent binding events with different affinity (Bier et al., 2013). This phenomenon is not unique. A recent study for the interactions between CLR01 and poly[ADP-ribose]polymerase (PARP-1) also show distinct multiple lysine recognition site (Wilch et al., 2017). More interestingly, such a recognition ability of CLR01 was considered to be an efficient inhibition method. Thanks to the cavity topology, there are other supramolecules that cannot specifically recognize the lysine residues, e.g., the para-sulfonato-calix[n] arenes (Perret et al., 2006). Indeed, in our binding free energy calculation, $\Delta G_{bind}^{calc}$ = −2.54 kcal·mol^−1^ for tweezer/K214 complex, which demonstrates a large difference from the experimental measurement of −6~-8 kcal·mol^−1^. Therefore, other lysine residues cannot be simply ignored if we want to investigate the interaction between tweezer and 14-3-3σ protein.

Another interesting issue is that the 14-3-3σ protein was shown to be active upon the dimerization, which exhibits a U-shape topology and the N-terminal helices constitute the dimer interface. This U-shape groove formed by either homo or hetero dimerization seems to be a conserved phenomenon in 14-3-3s (Jones et al., 1995; Messaritou et al., 2010). On the other hand, each monomer has its own canyon-like ligand binding groove. Such kind of structural characteristics have been suggested to be essential for 14-3-3s stability and functional activity, e.g., 14-3-3ζ (Messaritou et al., 2010). When 14-3-3s function as adaptor proteins in the functionally regulation of other proteins, the U-shaped groove serves as the key point via interaction with two motifs on single or multiple client proteins (Alblova et al., 2017; Chalupska et al., 2017; Karlberg et al., 2018). Inhibitors can thus be designed based on the following strategies, e.g., blocking client proteins moving in the binding groove or breaking the dimerization. If we carefully check the positions of surface lysine residues, we can see that the non-specific binding affinity seems to be quite suitable for supermolecular inhibitor design. As shown in Figure 1, the surface exposed K214 is situated at the beginning of the last C-terminal helix (α9), which is ready for forming an inclusion complex with the tweezer molecule. In fact, once the formation of the tweezer/K214 complex is completed, the binding channel of the external client protein will be clearly blocked (Bier et al., 2013). Meanwhile, the tweezer molecule also shows pretty strong interactions with some interfacial lysine residues on the basis of our MD simulation and binding free energy calculations. Such interactions can affect the dimerization without question. In other words, the non-specific recognition ability of the tweezer molecule against surface lysine residues could facilitate its inhibition to 14-3-3 proteins.

**Figure 7:**
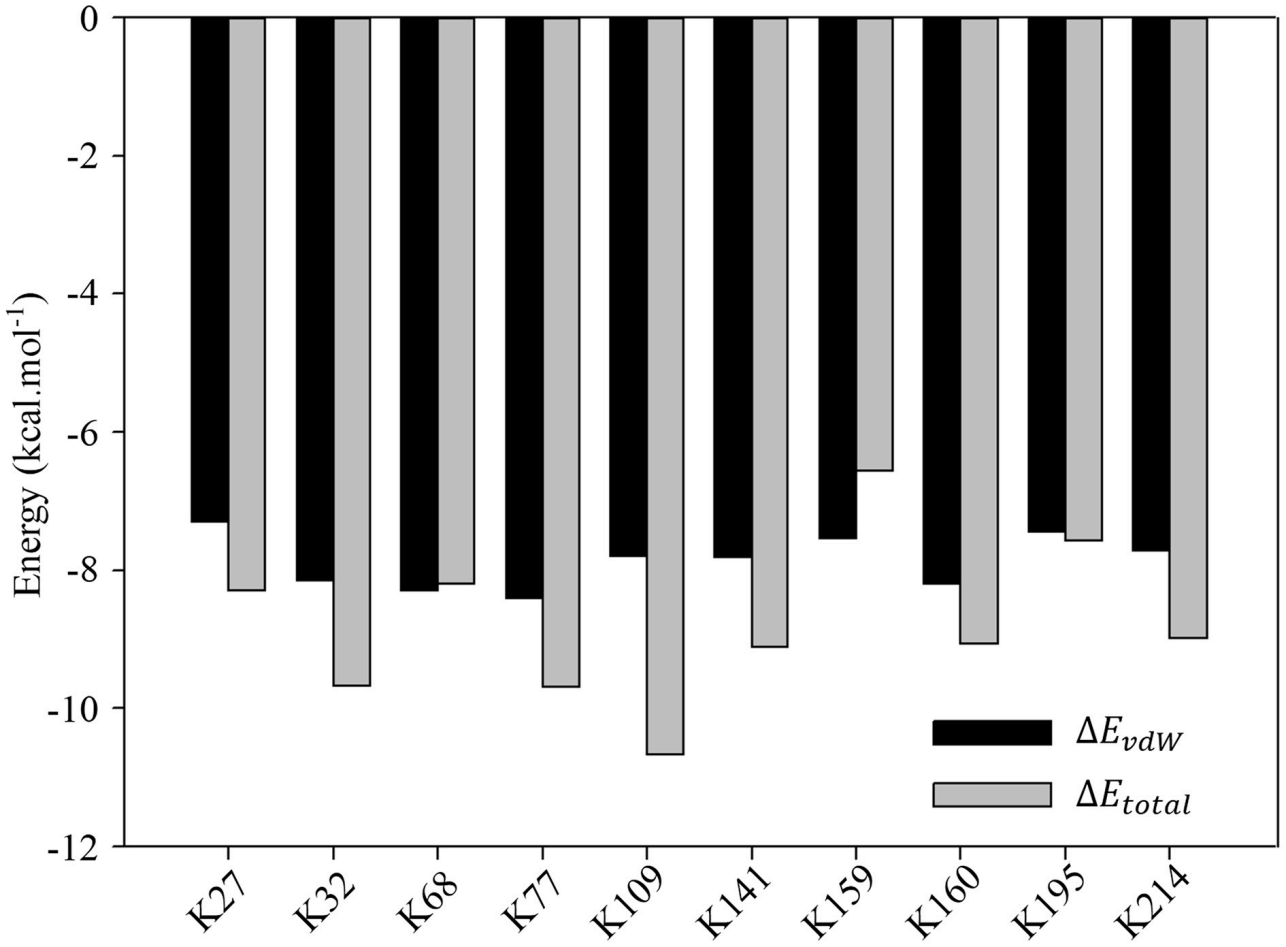
Comparison of *E*_*vdW*_ and *E*_*total*_ for K27, K32, K68, K77, K109, K141, K160, K195, and K214.

Nevertheless, detailed analyses of the ligand binding canyon of the monomer protein chain still stay at the core position. As we have mentioned above, including K214, there are 9 other lysine residues, which are ready for inclusion by the tweezer cavity. MD simulation can only provide some general idea of how the inhibitor bound to the lysine residues. It cannot give quantitative evaluation of contributions from surrounding residues. Based on our MM-GBSA calculations, further decomposition per residue calculations were carried out for all stable complexes. All results are summarized in Tables S1–S19. More importantly, if we carefully analyze all data presented there, we could find that the van der Waals interactions between lysine and tweezer dominate the binding energy. To illuminate this, we then plotted the comparison between calculated *E*_*vdW*_ and *E*_*total*_ in Figure 7. Obviously, if we do not consider other residues close to the lysine residue, the contributions to the binding free energy from lysine residues are generally very close.

Of course, our model used in this work only considered one monomer chain of 14-3-3σ protein. To get a more complete understanding of the inhibition mechanism, we might include the whole protein in the simulation in the future. On the other hand, since no external protein is included in our simulation, we cannot evaluate the detailed effects of the tweezer molecule in the regulation of protein-protein interactions. The simulation presented here can still shed some insights into the development of new inhibitors. For example, although the tweezer molecule shows its inhibition of the chaperone function of 14-3-3s against other proteins, such kind of non-specific recognition ability with lysine residues might cause some side effects. Our simulations also suggest that the nearby environment of those lysine sites could largely affect these kinds of interactions via space hindrances or other factors. On the other hand, those molecules with structural characteristics of polar/non-polar inner cavity and opposite polarity outside show particular functions in the regulation of the protein functions. Our MD simulations of the supramolecular complexes formed by cyclo[6]aramide and cytochrome c suggested that only 2 of 18 surface exposed lysine residues could be effectively recognized (Pan et al., 2018). Much larger size of the macrocyclic molecule could be one of the reasons to cause this effect. Therefore, the possible direction of the inhibitor design to 14-3-3 proteins should not just consider the features of molecular cavity, but also the size of the inhibitor.

## Conclusions

Small molecule regulation of PPI is currently one of the most promising and active fields in drug discovery and chemical biology. 14-3-3 proteins interact with hundreds of partner proteins and are involved in many physiological processes and diseases. Hence, strategies using small molecules to modulate these interactions have attracted extensive interests for years (Masters and Fu, 2001; Würtele et al., 2003; Ottmann et al., 2007a, 2009; Wu et al., 2010; Zhao et al., 2011; Arrendale et al., 2012; Bier et al., 2013; Thiel et al., 2013; Hartman and Hirsch, 2017; Ehlers et al., 2018; Stevers et al., 2018). One synthetic water-soluble belt-like supramolecular ligand, tweezer (CLR01), was specifically designed to inhibit the PPIs via strong binding affinity with those surface exposed lysine residues of 14-3-3σ. Therefore, in order to understand how the tweezer molecule regulates the interactions between 14-3-3σ and its partner proteins, the possible recognition behavior should be identified first.

We, in this work, systematically investigated the recognition ability of all surface lysine residues by the tweezer molecule using classical MD simulations and corresponding binding free energy calculations. A total 10 of 17 lysine residues were found to form the possible inclusion complexes with this inhibitor. Major contact comes from interactions between the long alkyl chain of lysine and the cavity formed by the norbornadiene and benzene rings of the tweezer molecule. In addition, the stable ion pair formed between the phosphate group of the inhibitor and positive charge residues on the surface of protein could provide additional contributions. Our simulation clearly indicates there is no preferential lysine recognition site for the tweezer molecule. We can locate all the possible binding sites either on the protein surface or at the dimeric interfacial region. Such recognition features might facilitate its function in the regulation of possible 14-3-3σ involved PPIs. On the other hand, this non-specific recognition could also cause unexpected side effects. Finally, to completely understand the effects of the inhibitor in the PPI, we have to include the second protein in our simulation, e.g., ExoS. Simulation work to this direction is underway in our lab.

## Author Contributions

MS and DX contributed conception and design of the study, and performed the statistical analysis, and wrote the manuscript. All authors contributed to manuscript revision, read and approved the submitted version.

### Conflict of Interest Statement

The authors declare that the research was conducted in the absence of any commercial or financial relationships that could be construed as a potential conflict of interest.
