# Supplementary material for: Molecular Dynamics Investigations Suggest a Non-specific Recognition Strategy of 14-3-3σ Protein by Tweezer: Implication for the Inhibition Mechanism
Source: Front Chem. 2019 Apr 17;7:237. doi: 10.3389/fchem.2019.00237 (PMC6478809; doi:10.3389/fchem.2019.00237)
Supplement: Supplementary file 1 [file Data_Sheet_1.docx]

**Supporting Information**

**Molecular Dynamics Investigations Suggest a Non-Specific Recognition Strategy of 14-3-3σ Protein by Tweezer: Implication for the Inhibition Mechanism**

Mingsong Shi and Dingguo Xu*

*MOE Key Laboratory of Green Chemistry, College of Chemistry, Sichuan University, Chengdu, Sichuan, P. R. China 610064*

* To whom correspondence should be addressed: dgxu@scu.edu.cn (D.X), Tel: 86-28-85406156

**Contents**

[Figure S1. The selected structure of the tweezer/K27 after docking simulation. S1](#_Toc527805452)

[Figure S2. The selected structure of the tweezer/K32 after docking simulation. S2](#_Toc527805453)

[Figure S3. The selected structure of the tweezer/K49 after docking simulation. S3](#_Toc527805454)

[Figure S4. Snapshots of the tweezer/K49 complex along the dynamics simulation time. S4](#_Toc527805455)

[Figure S5. The selected structure of the tweezer/K68 after docking simulaiton. S5](#_Toc527805456)

[Figure S6. 2-D Protein-ligand interaction depictions of tweezer/K68 complex. S6](#_Toc527805457)

[Figure S7. The selected structure of the tweezer/K77 after docking simulation. S7](#_Toc527805458)

[Figure S8. The selected structure of the tweezer/K109 after docking simulation. S8](#_Toc527805459)

[Figure S9. The selected structure of the tweezer/K122 after docking simulation. S9](#_Toc527805460)

[Figure S10. The selected structure of the tweezer/K124 after docking simulation. S10](#_Toc527805461)

[Figure S11. The selected structure of the tweezer/K140 after docking simulation. S11](#_Toc527805462)

[Figure S12. Snapshots of tweezer/K140 complex along the dynamic simulation time. S12](#_Toc527805463)

[Figure S13. The selected structure of the tweezer/K141 after docking simulation. S13](#_Toc527805464)

[Figure S14. The selected structure of the tweezer/K159 after docking simulation. S14](#_Toc527805465)

[Figure S15. The selected structure of the tweezer/K160 after docking simulation. S15](#_Toc527805466)

[Figure S16. The selected structure of the tweezer/K195 after docking simulation. S16](#_Toc527805467)

[Figure S17. Snapshots of complexes for I-type lysines along the dynamic simulation time for I-type lysine site. S17](#_Toc527805468)

[Figure S18. Snapshots of the tweezer/K complexes along the dynamic simulation time for III-type lysine site except K214. S18](#_Toc527805469)

[Figure S19. Snapshots of the tweezer/K binding models along the dynamic simulation time for IV-type lysine site. S19](#_Toc527805470)

[Table S1. Binding free energy decomposition per residue for tweezer/K214. S20](#_Toc527805471)

[Table S2. Binding free energy for tweezer/K27. S21](#_Toc527805472)

[Table S3. Binding free energy decomposition per residue for tweezer/K27. S22](#_Toc527805473)

[Table S4. Binding free energy for tweezer/K32. S23](#_Toc527805474)

[Table S5. Binding free energy decomposition per residue for tweezer/K32. S24](#_Toc527805475)

[Table S6. Binding free energy for tweezer/K68. S25](#_Toc527805476)

[Table S7. Binding free energy decomposition per residue for tweezer/K68. S26](#_Toc527805477)

[Table S8. Binding free energy for tweezer/K77. S27](#_Toc527805478)

[Table S9. Binding free energy decomposition per residue for tweezer/K77. S28](#_Toc527805479)

[Table S10. Binding free energy for tweezer/K109. S29](#_Toc527805480)

[Table S11. Binding free energy decomposition per residue for tweezer/K109. S30](#_Toc527805481)

[Table S12. Binding free energy for tweezer/K141. S31](#_Toc527805482)

[Table S13. Binding free energy decomposition per residue for tweezer/K141. S32](#_Toc527805483)

[Table S14. Binding free energy for tweezer/K159. Energies are in kcal/mol. S33](#_Toc527805484)

[Table S15. Binding free energy decomposition per residue for tweezer/K159. S34](#_Toc527805485)

[Table S16. Binding free energy for tweezer/K160. Energies are in kcal/mol. S35](#_Toc527805486)

[Table S17. Binding free energy decomposition per residue for tweezer/K160. S36](#_Toc527805487)

[Table S18. Binding free energy for tweezer/K195. S37](#_Toc527805488)

[Table S19. Binding free energy decomposition per residue for tweezer/K195. S38](#_Toc527805489)

Table S20. Binding free energy for tweezer/K214 from docking result. S39


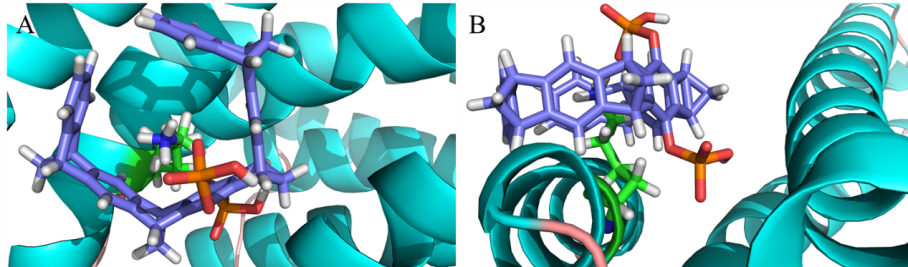


# Figure S1. The selected structure of the tweezer/K27 after docking simulation. (A) top view, (B) side view.


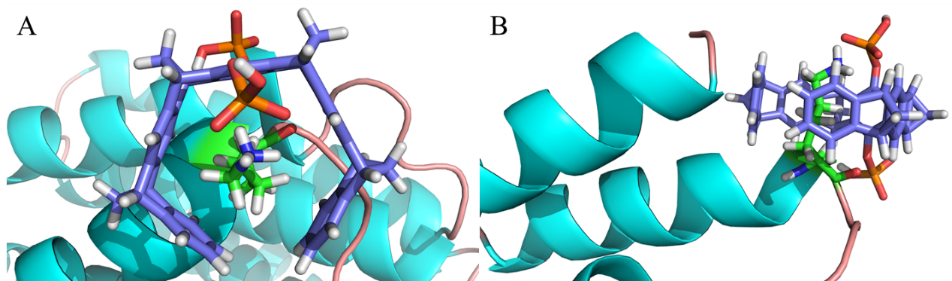


# Figure S2. The selected structure of the tweezer/K32 after docking simulation. (A) top view, (B) side view.


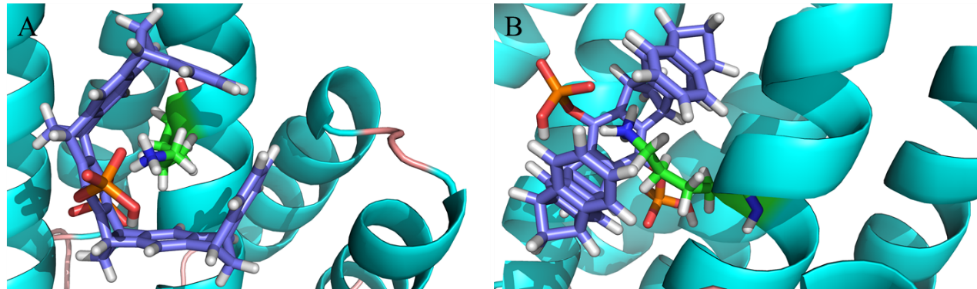


# Figure S3. The selected structure of the tweezer/K49 after docking simulation. (A) top view, (B) side view.


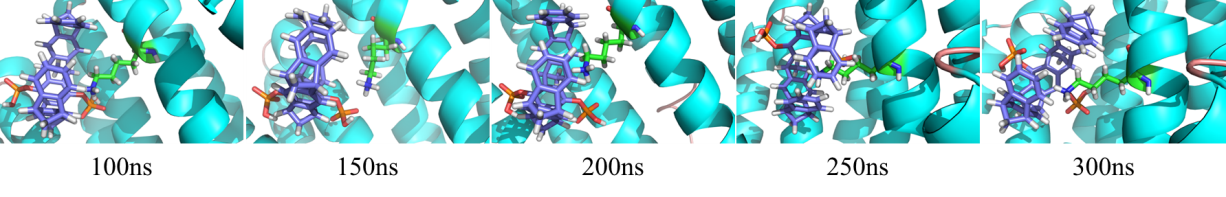


# Figure S4. Snapshots of the tweezer/K49 complex along the dynamics simulation time. For clarity, all water molecules have been removed.


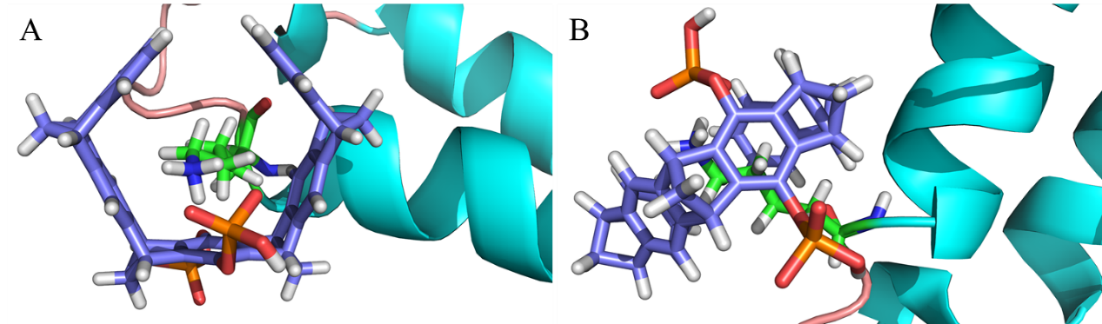


# Figure S5. The selected structure of the tweezer/K68 after docking simulaiton. (A) top view, (B) side view.


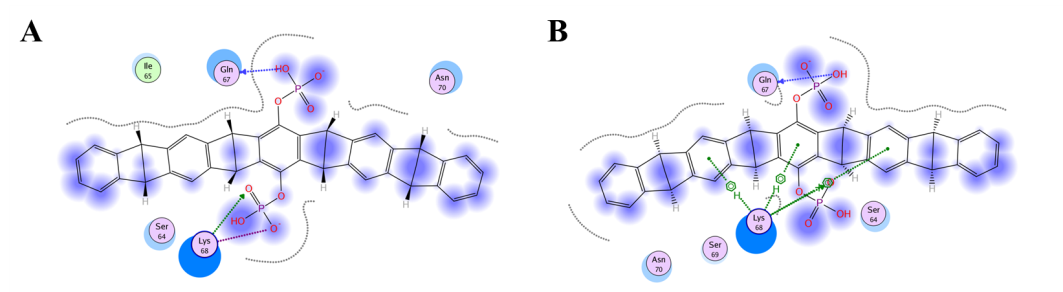


# Figure S6. 2-D Protein-ligand interaction depictions of tweezer/K68 complex. (A) shows the 2-D protein-ligand interaction depictions for docked complex and (B) for the end of MD simulation (300ns)


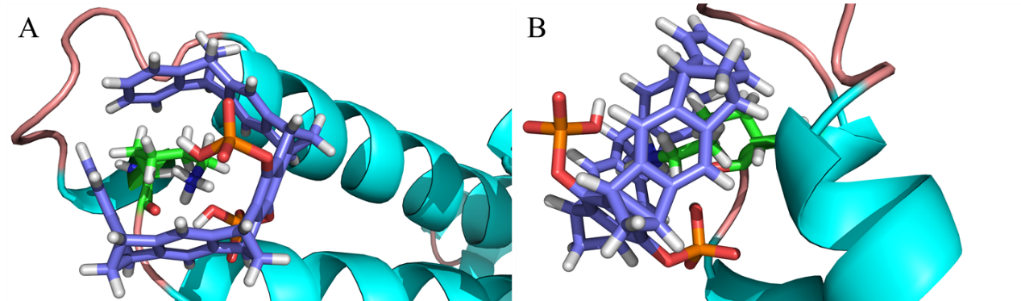


# Figure S7. The selected structure of the tweezer/K77 after docking simulation. (A) top view, (B) side view.


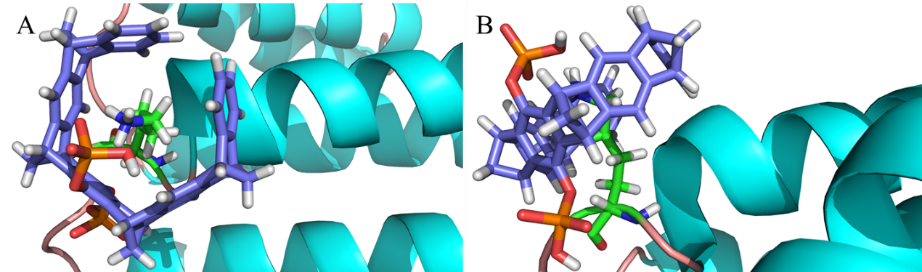


# Figure S8. The selected structure of the tweezer/K109 after docking simulation. (A) top view, (B) side view.


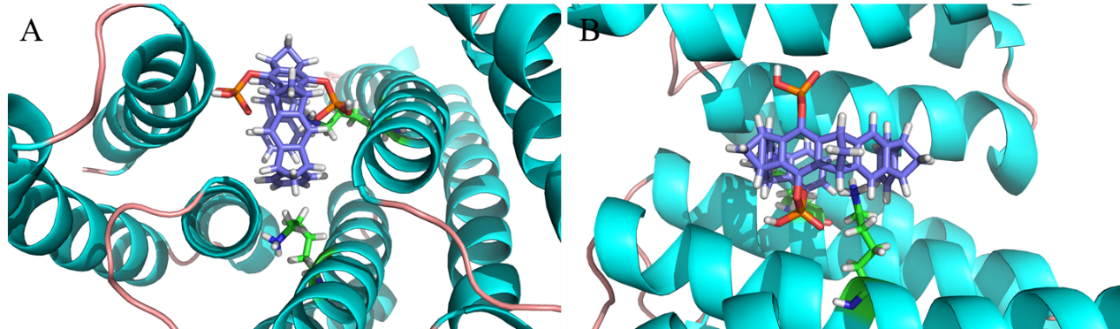


# Figure S9. The selected structure of the tweezer/K122 after docking simulation. (A) top view, (B) side view.


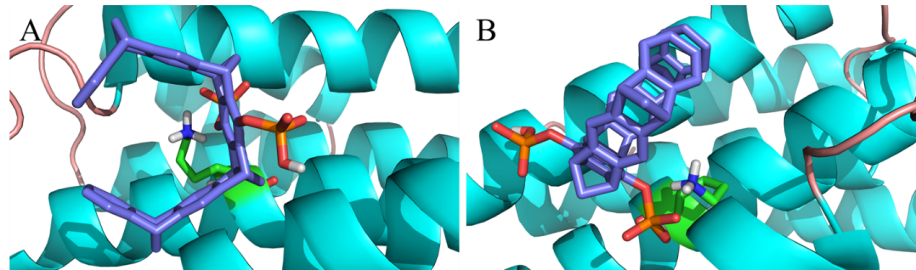


# Figure S10. The selected structure of the tweezer/K124 after docking simulation. (A) top view, (B) side view.


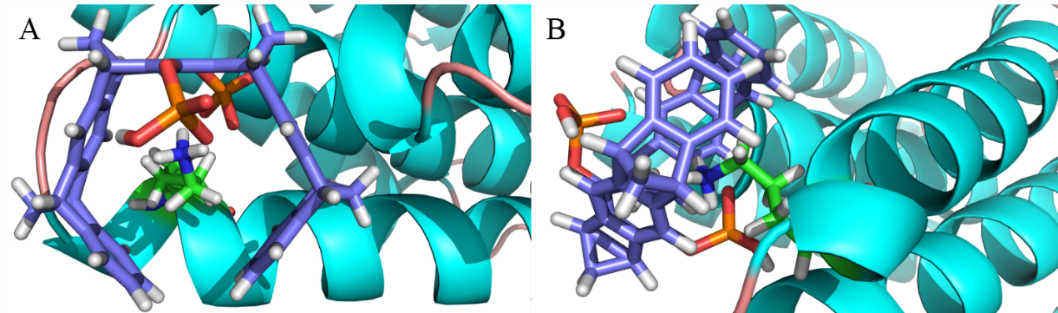


# Figure S11. The selected structure of the tweezer/K140 after docking simulation. (A) top view, (B) side view.

**
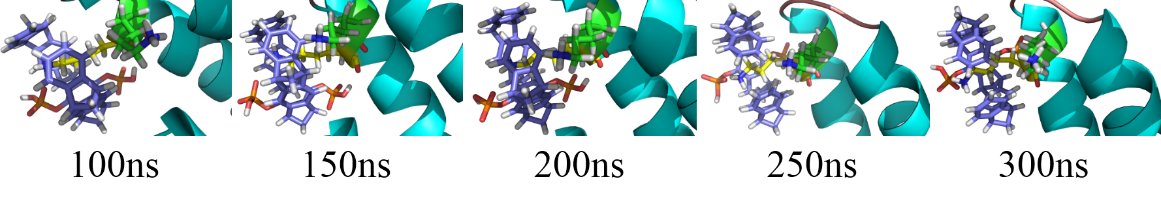
**

# Figure S12. Snapshots of tweezer/K140 complex along the dynamic simulation time. For clarity, the water molecules have been removed.


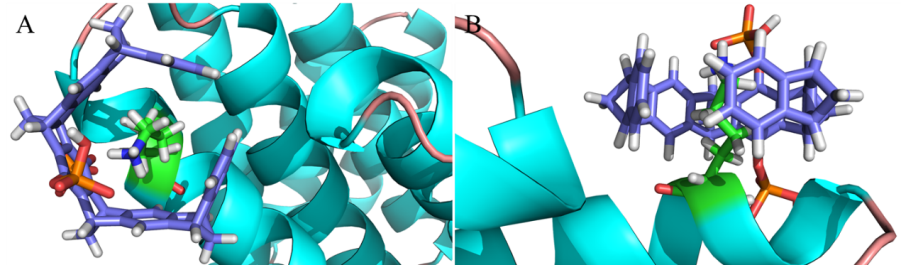


# Figure S13. The selected structure of the tweezer/K141 after docking simulation. (A) top view, (B) side view.


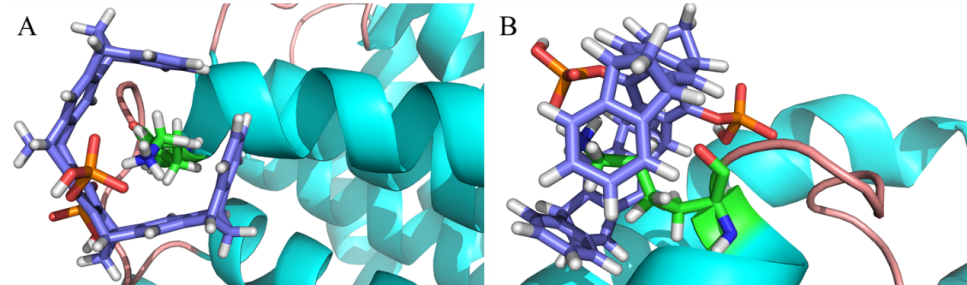


# Figure S14. The selected structure of the tweezer/K159 after docking simulation. (A) top view, (B) side view.


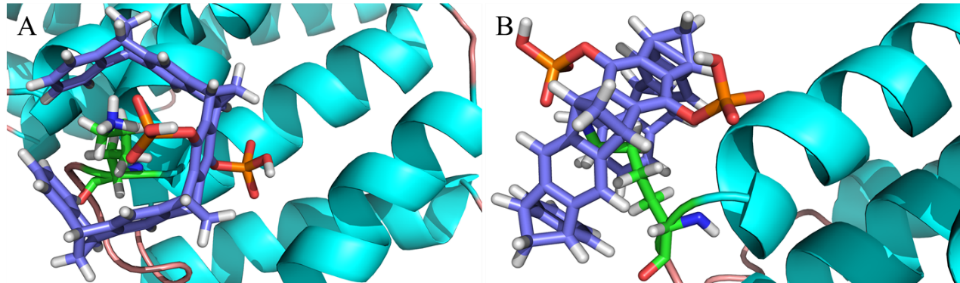


# Figure S15. The selected structure of the tweezer/K160 after docking simulation. (A) top view, (B) side view.


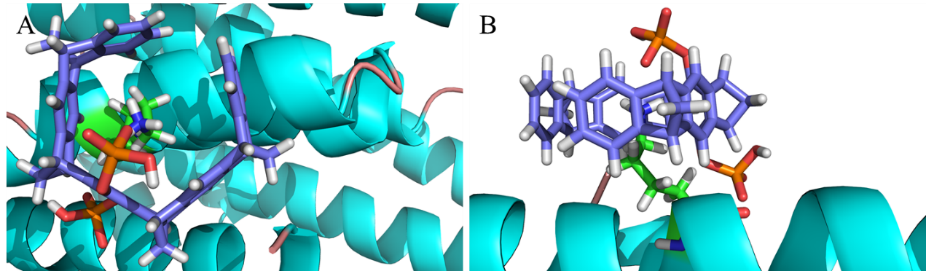


# Figure S16. The selected structure of the tweezer/K195 after docking simulation. (A) top view, (B) side view.


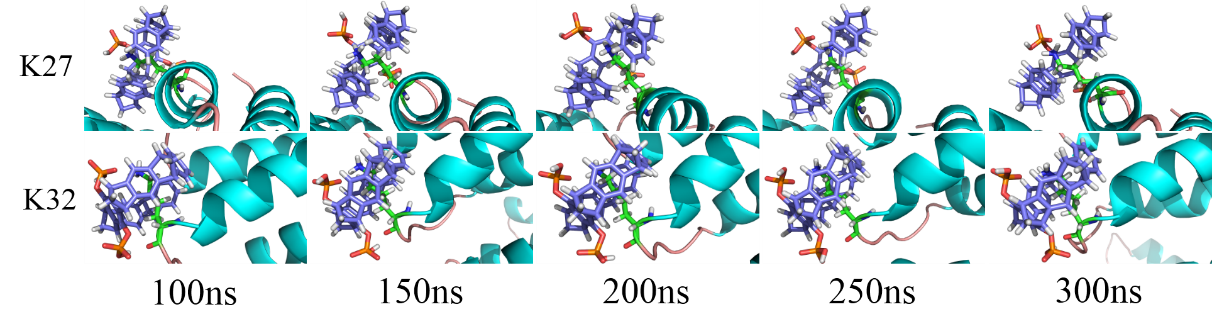


**Figure S17.** Snapshots of complexes for I-type lysines along the dynamic simulation time for I-type lysine site. For clarity, the water molecules have been removed..


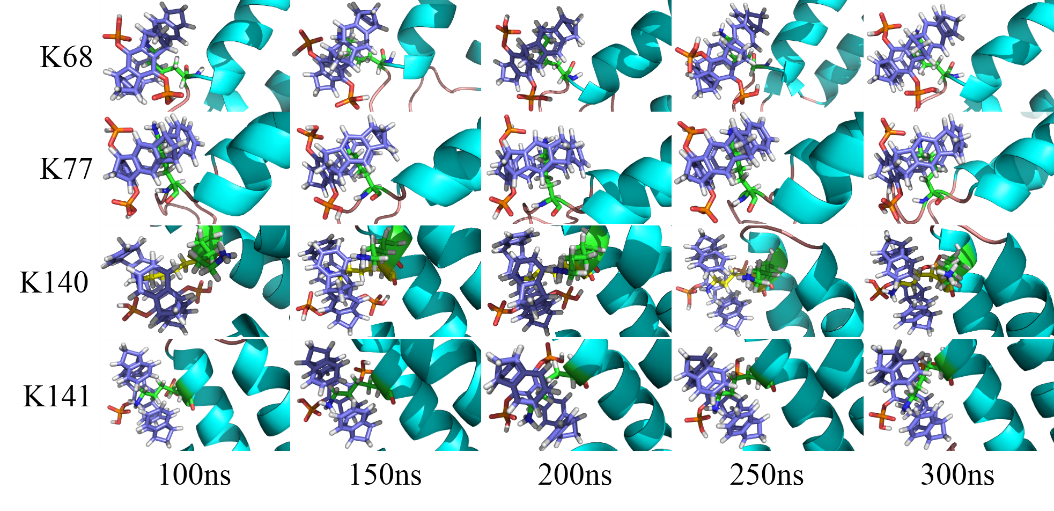


**Figure S18.** Snapshots of the tweezer/K complexes along the dynamic simulation time for III-type lysine site except K214. For clarity, the water molecules have been removed.


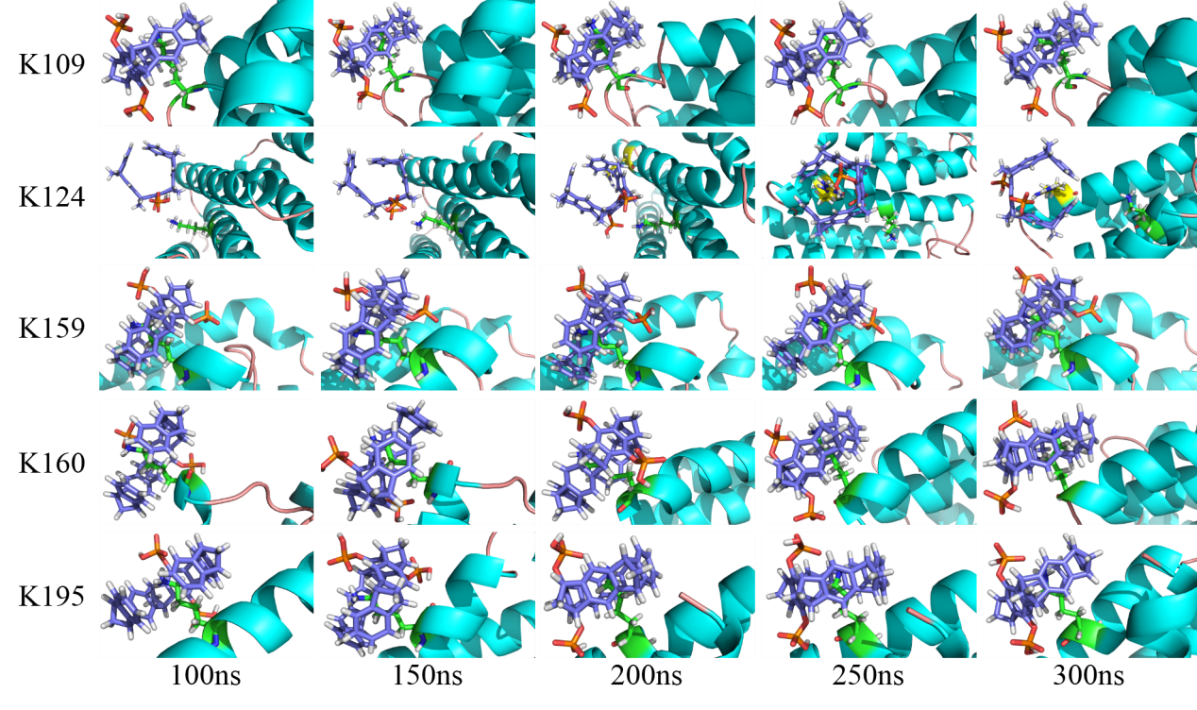


**Figure S19.** Snapshots of the tweezer/K binding models along the dynamic simulation time for IV-type lysine site. For clarity, the water molecules have been removed.

**Table S1.** Binding free energy decomposition per residue for tweezer/K214. For every residue decomposed free energy to electrostatic interaction, van der Walls interaction, polar solvation energy and non-polar solvation energy. Only the residues contributing significantly to the tweezer-14-3-3σ complex are highlighted here. Energies are in kcal/mol.

| Residues | $\Delta E_{vdW}$ | $\Delta E_{ele}$ | $\Delta E_{sol,GB}$ | $\Delta E_{sol,np}$ | $S\Delta E_{subtotal}$ | $B\Delta E_{subtotal}$ | $\Delta E_{total}$ |
| --- | --- | --- | --- | --- | --- | --- | --- |
| Y213 | -1.58 | -0.67 | 1.14 | -0.21 | -0.85 | -0.46 | -1.31 |
| K214 | -7.72 | -78.37 | 78.35 | -1.24 | -7.70 | -1.27 | -8.98 |
| D215 | -0.83 | 42.26 | -38.95 | -0.17 | 2.41 | -0.10 | 2.31 |
| T217 | -1.23 | -1.98 | 2.28 | -0.15 | -0.82 | -0.27 | -1.09 |
| L218 | -1.87 | -1.68 | 1.82 | -0.32 | -1.80 | -0.26 | -2.05 |

# Table S2. Binding free energy for tweezer/K27. Energies are in kcal/mol.

|  | complex | receptor | ligand | delta |
| --- | --- | --- | --- | --- |
| $E_{\mathrm{vdW}}$ | -1873.84(21.25) | -1814.36(21.01) | -20.60(1.19) | -38.88(3.14) |
| $E_{\mathrm{ele}}$ | -15972.81(124.24) | -16247.83(121.89) | 148.77(3.75) | 126.25(25.25) |
| $E_{\mathrm{GB}}$ | -4558.76(109.48) | -4258.32(108.42) | -181.66(2.05) | -118.78(23.20) |
| $E_{\mathrm{surf}}$ | 100.86(2.10) | 100.81(2.08) | 4.68(0.08) | -4.63(0.28) |
| $G_{\mathrm{gas}}$ | -1707.06(126.50) | -1894.92(125.57) | 100.53(6.67) | 87.33(24.96) |
| $G_{\mathrm{solv}}$ | -4457.89(108.59) | -4157.51(107.47) | -176.98(2.05) | -123.41(23.15) |
| $E_{\mathrm{gas}}+G_{\mathrm{sol}}$ | -6164.96(47.41) | -6052.43(46.85) | -76.45(6.58) | -36.08(3.73) |
| $TS_{\mathrm{total}}$ | 2628.06(8.90) | 2580.12(8.83) | 71.24(0.08) | -23.30(3.85) |
| $\Delta G_{\mathrm{bind}}^{\mathrm{cal}}$ |  |  |  | -12.78 |

# Table S3. Binding free energy decomposition per residue for tweezer/K27. Energies are in kcal/mol.

| Residues | $\Delta E_{vdW}$ | $\Delta E_{ele}$ | $\Delta E_{sol,GB}$ | $\Delta E_{sol,np}$ | $S\Delta E_{subtotal}$ | $B\Delta E_{subtotal}$ | $\Delta E_{total}$ |
| --- | --- | --- | --- | --- | --- | --- | --- |
| A23 | -0.78 | 1.14 | -0.86 | -0.07 | -0.44 | -0.13 | -0.57 |
| A24 | -1.09 | 1.35 | -0.96 | -0.21 | -0.72 | -0.19 | -0.91 |
| K27 | -7.31 | -77.89 | 78.23 | -1.32 | -7.12 | -1.17 | -8.29 |
| G28 | -0.62 | -1.30 | 1.35 | -0.13 | -0.17 | -0.53 | -0.71 |
| E31 | -0.33 | 36.53 | -35.47 | -0.06 | 0.58 | 0.09 | 0.67 |
| Y48 | -0.96 | -0.39 | 0.89 | -0.08 | -0.44 | -0.10 | -0.54 |
| V51 | -1.44 | -0.94 | 0.87 | -0.29 | -1.65 | -0.16 | -1.80 |
| V95 | -0.45 | 0.79 | -0.80 | -0.06 | -0.39 | -0.13 | -0.51 |
| T98 | -1.24 | 0.95 | -0.91 | -0.28 | -1.18 | -0.31 | -1.48 |
| V99 | -0.54 | 0.42 | -0.37 | -0.04 | -0.43 | -0.10 | -0.54 |
| L102 | -1.64 | -0.72 | 0.73 | -0.27 | -1.72 | -0.18 | -1.90 |
| H106 | -0.28 | -47.43 | 45.25 | -0.16 | -2.59 | -0.04 | -2.63 |

# Table S4. Binding free energy for tweezer/K32. Energies are in kcal/mol.

|  | complex | receptor | ligand | delta |
| --- | --- | --- | --- | --- |
| $E_{\mathrm{vdW}}$ | -1887.22(21.22) | -1829.76(21.15) | -20.38(1.06) | -37.08(3.02) |
| $E_{\mathrm{ele}}$ | -15880.61(148.87) | -16118.84(142.62) | 148.32(3.40) | 89.91(19.48) |
| $E_{\mathrm{GB}}$ | -4636.23(122.36) | -4375.25(116.24) | -181.84(1.96) | -79.15(18.84) |
| $E_{\mathrm{surf}}$ | 99.41(1.82) | 98.78(1.84) | 4.70(0.07) | -4.07(0.24) |
| $G_{\mathrm{gas}}$ | -1644.70(144.65) | -1796.88(138.69) | 99.39(6.42) | 52.79(19.30) |
| $G_{\mathrm{solv}}$ | -4536.82(121.81) | -4276.47(115.61) | -177.14(1.97) | -83.22(18.83) |
| $E_{\mathrm{gas}}+G_{\mathrm{sol}}$ | -6181.53(49.80) | -6073.35(49.52) | -77.75(6.36) | -30.43(2.76) |
| $TS_{\mathrm{total}}$ | 2628.47(7.82) | 2578.27(7.60) | 71.34(0.05) | -21.14(2.84) |
| $\Delta G_{\mathrm{bind}}^{\mathrm{cal}}$ |  |  |  | -9.29 |

# Table S5. Binding free energy decomposition per residue for tweezer/K32. Energies are in kcal/mol.

| Residues | $\Delta E_{vdW}$ | $\Delta E_{ele}$ | $\Delta E_{sol,GB}$ | $\Delta E_{sol,np}$ | $S\Delta E_{subtotal}$ | $B\Delta E_{subtotal}$ | $\Delta E_{total}$ |
| --- | --- | --- | --- | --- | --- | --- | --- |
| M1 | -0.98 | -29.95 | 31.83 | -0.18 | -0.46 | 1.18 | 0.72 |
| R3 | -2.26 | -27.10 | 27.44 | -0.24 | -1.46 | -0.70 | -2.16 |
| L6 | -0.76 | -1.25 | 1.27 | -0.11 | -0.77 | -0.07 | -0.84 |
| E31 | -1.46 | 35.75 | -34.55 | -0.24 | -0.46 | -0.05 | -0.51 |
| K32 | -8.16 | -75.69 | 75.54 | -1.37 | -8.08 | -1.61 | -9.68 |

# Table S6. Binding free energy for tweezer/K68. Energies are in kcal/mol.

|  | complex | receptor | ligand | delta |
| --- | --- | --- | --- | --- |
| $E_{\mathrm{vdW}}$ | -1880.92(19.53) | -1829.18(19.55) | -20.06(1.31) | -31.68(2.70) |
| $E_{\mathrm{ele}}$ | -15889.59(114.44) | -16222.08(115.71) | 147.88(3.69) | 184.61(19.78) |
| $E_{\mathrm{GB}}$ | -4666.15(94.24) | -4309.49(95.87) | -182.16(2.16) | -174.50( 17.61) |
| $E_{\mathrm{surf}}$ | 99.85(1.67) | 98.36(1.68) | 4.72(0.09) | -3.23(0.20) |
| $G_{\mathrm{gas}}$ | -1618.41(113.62) | -1871.03(115.30) | 99.72(6.60) | 152.90(19.25) |
| $G_{\mathrm{solv}}$ | -4566.30(93.54) | -4211.13(95.14) | -177.44(2.15) | -177.73(17.60) |
| $E_{\mathrm{gas}}+G_{\mathrm{sol}}$ | -6184.72(46.76) | -6082.16(46.70) | -77.72(6.43) | -24.84(3.06) |
| $TS_{\mathrm{total}}$ | 2626.02(7.64) | 2574.75(7.96) | 71.27(0.15) | -20.00(2.32) |
| $\Delta G_{\mathrm{bind}}^{\mathrm{cal}}$ |  |  |  | -4.84 |

# Table S7. Binding free energy decomposition per residue for tweezer/K68. Energies are in kcal/mol.

| Residues | $\Delta E_{vdW}$ | $\Delta E_{ele}$ | $\Delta E_{sol,GB}$ | $\Delta E_{sol,np}$ | $S\Delta E_{subtotal}$ | $B\Delta E_{subtotal}$ | $\Delta E_{total}$ |
| --- | --- | --- | --- | --- | --- | --- | --- |
| S64 | -1.26 | 1.36 | -0.68 | -0.23 | -0.45 | -0.36 | -0.81 |
| I65 | -0.74 | 1.20 | -1.15 | -0.08 | -0.49 | -0.28 | -0.77 |
| Q67 | -1.77 | 1.14 | 0.05 | -0.35 | -0.82 | -0.09 | -0.92 |
| K68 | -8.29 | -71.66 | 73.02 | -1.27 | -7.39 | -0.81 | -8.20 |
| N70 | -1.50 | 3.00 | -1.85 | -0.30 | -0.39 | -0.26 | -0.65 |
| E76 | -0.23 | 26.08 | -25.24 | -0.04 | 0.54 | 0.02 | 0.57 |

# Table S8. Binding free energy for tweezer/K77. Energies are in kcal/mol.

|  | complex | receptor | ligand | delta |
| --- | --- | --- | --- | --- |
| $E_{\mathrm{vdW}}$ | -1883.28(21.56) | -1820.35(21.70) | -19.97(1.19) | -42.96(3.18) |
| $E_{\mathrm{ele}}$ | -15801.89(115.06) | -16167.51(113.42) | 147.40(3.72) | 218.22(17.39) |
| $E_{\mathrm{GB}}$ | -4702.76(96.01) | -4316.78(95.56) | -181.37(2.09) | -204.61(15.70) |
| $E_{\mathrm{surf}}$ | 99.72(2.21) | 99.42(2.25) | 4.73(0.08) | -4.43(0.26) |
| $G_{\mathrm{gas}}$ | -1578.00(114.54) | -1853.00(113.81) | 99.78(6.42) | 175.22(17.09) |
| $G_{\mathrm{solv}}$ | -4603.04(95.17) | -4217.35(94.65) | -176.65(2.09) | -209.04(15.71) |
| $E_{\mathrm{gas}}+G_{\mathrm{sol}}$ | -6181.04(47.65) | -6070.35(46.96) | -76.87(6.30) | -33.82(3.36) |
| $TS_{\mathrm{total}}$ | 2630.18(8.49) | 2579.90(8.43) | 71.26(0.10) | -20.98(3.20) |
| $\Delta G_{\mathrm{bind}}^{\mathrm{cal}}$ |  |  |  | -12.84 |

# Table S9. Binding free energy decomposition per residue for tweezer/K77. Energies are in kcal/mol.

| Residues | $\Delta E_{vdW}$ | $\Delta E_{ele}$ | $\Delta E_{sol,GB}$ | $\Delta E_{sol,np}$ | $S\Delta E_{subtotal}$ | $B\Delta E_{subtotal}$ | $\Delta E_{total}$ |
| --- | --- | --- | --- | --- | --- | --- | --- |
| Q67 | -1.33 | 0.99 | 0.05 | -0.21 | -0.39 | -0.11 | -0.50 |
| E75 | -2.14 | 33.87 | -31.97 | -0.35 | -0.55 | -0.04 | -0.59 |
| E76 | -1.92 | 31.34 | -31.23 | -0.16 | -0.50 | -1.48 | -1.97 |
| K77 | -8.41 | -72.34 | 72.41 | -1.34 | -8.22 | -1.47 | -9.69 |
| P79 | -1.21 | -0.70 | 0.78 | -0.18 | -0.96 | -0.34 | -1.31 |
| R82 | -2.29 | -31.73 | 32.14 | -0.36 | -2.00 | -0.24 | -2.23 |

**Table S10.** Binding free energy for tweezer/K109. Energies are in kcal/mol.

|  | complex | receptor | ligand | delta |
| --- | --- | --- | --- | --- |
| $E_{\mathrm{vdW}}$ | -1875.98(19.86) | -1825.02(19.44) | -20.42(1.08) | -30.54(3.10) |
| $E_{\mathrm{ele}}$ | -15924.71(125.64) | -16199.55(123.39) | 148.43(3.46) | 126.41(22.31) |
| $E_{\mathrm{GB}}$ | -4592.20(109.12) | -4289.08(108.34) | -181.23(2.01) | -121.90(17.93) |
| $E_{\mathrm{surf}}$ | 100.01(1.85) | 98.90(1.87) | 4.68(0.08) | -3.57 (0.26) |
| $G_{\mathrm{gas}}$ | -1682.11(124.82) | -1877.40(122.57) | 99.46(6.62) | 95.83(21.56) |
| $G_{\mathrm{solv}}$ | -4492.19(108.43) | -4190.18(107.61) | -176.54(2.02) | -125.47(17.89) |
| $E_{\mathrm{gas}}+G_{\mathrm{sol}}$ | -6174.30(45.98) | -6067.57(45.02) | -77.09(6.51) | -29.64(6.36) |
| $TS_{\mathrm{total}}$ | 2628.49(8.36) | 2580.27(8.30) | 71.24(0.04) | -23.02(3.05) |
| $\Delta G_{\mathrm{bind}}^{\mathrm{cal}}$ |  |  |  | -6.62 |

# Table S11. Binding free energy decomposition per residue for tweezer/K109. Energies are in kcal/mol.

| Residues | $\Delta E_{vdW}$ | $\Delta E_{ele}$ | $\Delta E_{sol,GB}$ | $\Delta E_{sol,np}$ | $S\Delta E_{subtotal}$ | $B\Delta E_{vdW}$ | $\Delta E_{total}$ |
| --- | --- | --- | --- | --- | --- | --- | --- |
| D104 | -1.27 | 30.68 | -28.57 | -0.22 | -0.03 | 0.65 | 0.62 |
| I108 | -1.36 | 2.14 | -1.48 | -0.16 | -0.93 | 0.07 | -0.87 |
| K109 | -7.80 | -81.65 | 80.03 | -1.25 | -7.46 | -3.21 | -10.67 |
| E110 | -1.77 | 32.20 | -31.23 | -0.35 | 0.03 | -1.18 | -1.15 |
| A111 | -0.31 | -1.72 | 1.49 | -0.01 | -0.15 | -0.40 | -0.55 |
| R117 | -0.32 | -49.86 | 46.61 | -0.21 | -3.70 | -0.08 | -3.78 |

**Table S12.** Binding free energy for tweezer/K141. Energies are in kcal/mol.

|  | complex | receptor | ligand | delta |
| --- | --- | --- | --- | --- |
| $E_{\mathrm{vdW}}$ | -1887.27(20.01) | -1824.87(19.58) | -20.08(1.32) | -42.32(3.46) |
| $E_{\mathrm{ele}}$ | -15868.66(117.43) | -16191.91(116.95) | 148.19(3.74) | 175.07(19.60) |
| $E_{\mathrm{GB}}$ | -4663.85(98.52) | -4317.98(98.72) | -181.73(1.95) | -164.15(18.23) |
| $E_{\mathrm{surf}}$ | 99.73(1.74) | 99.51(1.71) | 4.67(0.07) | -4.45(0.31) |
| $G_{\mathrm{gas}}$ | -1621.11(109.96) | -1854.49(110.24) | 100.66(6.60) | 132.71(19.90) |
| $G_{\mathrm{solv}}$ | -4564.12(97.68) | -4218.46(97.92) | -177.06(1.95) | -168.60(18.15) |
| $E_{\mathrm{gas}}+G_{\mathrm{sol}}$ | -6185.24(44.13) | -6072.95(43.61) | -76.39(6.47) | -35.89 (3.90) |
| $TS_{\mathrm{total}}$ | 2629.60(7.73) | 2581.49(8.04) | 71.20(0.13) | -23.09(3.69) |
| $\Delta G_{\mathrm{bind}}^{\mathrm{cal}}$ |  |  |  | -12.80 |

# Table S13. Binding free energy decomposition per residue for tweezer/K141. Energies are in kcal/mol.

| Residues | $\Delta E_{vdW}$ | $\Delta E_{ele}$ | $\Delta E_{sol,GB}$ | $\Delta E_{sol,np}$ | $S\Delta E_{subtotal}$ | $B\Delta E_{subtotal}$ | $\Delta E_{total}$ |
| --- | --- | --- | --- | --- | --- | --- | --- |
| G137 | -0.89 | 0.61 | -0.09 | -0.15 | -0.23 | -0.30 | -0.53 |
| D138 | -1.63 | 38.64 | -37.44 | -0.22 | 0.05 | -0.70 | -0.65 |
| K140 | -1.20 | -26.74 | 27.17 | -0.14 | -0.54 | -0.35 | -0.89 |
| K141 | -7.81 | -78.04 | 77.95 | -1.21 | -8.07 | -1.04 | -9.11 |
| R142 | -0.99 | -39.16 | 38.30 | -0.15 | -1.55 | -0.44 | -1.99 |
| I144 | -1.55 | -1.46 | 1.48 | -0.13 | -1.28 | -0.38 | -1.66 |
| R148 | -1.24 | -33.92 | 34.34 | -0.20 | -0.93 | -0.08 | -1.01 |
| A184 | -1.43 | 0.83 | -0.48 | -0.18 | -0.77 | -0.48 | -1.25 |

**Table S14.** Binding free energy for tweezer/K159. Energies are in kcal/mol.

|  | complex | receptor | ligand | delta |
| --- | --- | --- | --- | --- |
| $E_{\mathrm{vdW}}$ | -1895.81(20.11) | -1839.13(19.84) | -19.99(1.35) | -36.69(3.43) |
| $E_{\mathrm{ele}}$ | -15798.21(160.44) | -16147.20(159.80) | 147.85(3.79) | 201.13(23.42) |
| $E_{\mathrm{GB}}$ | -4730.83(129.51) | -4356.87(129.03) | -181.45(2.19) | -192.51(21.09) |
| $E_{\mathrm{surf}}$ | 97.06(1.81) | 96.91(1.79) | 4.69(0.08) | -4.54(0.33) |
| $G_{\mathrm{gas}}$ | -1562.34(149.83) | -1827.04(148.81) | 100.29(6.82) | 164.41(22.87) |
| $G_{\mathrm{solv}}$ | -4633.78(128.55) | -4259.96(128.09) | -176.76(2.19) | -197.05(21.11) |
| $E_{\mathrm{gas}}+G_{\mathrm{sol}}$ | -6196.12(47.97) | -6087.00(47.13) | -76.47(6.62) | -32.65(3.88) |
| $TS_{\mathrm{total}}$ | 2623.50(8.64) | 2575.66(9.15) | 71.24(0.10) | -23.39(2.66) |
| $\Delta G_{\mathrm{bind}}^{\mathrm{cal}}$ |  |  |  | -9.26 |

# Table S15. Binding free energy decomposition per residue for tweezer/K159. Energies are in kcal/mol.

| Residues | $\Delta E_{vdW}$ | $\Delta E_{ele}$ | $\Delta E_{sol,GB}$ | $\Delta E_{sol,np}$ | $S\Delta E_{subtotal}$ | $B\Delta E_{subtotal}$ | $\Delta E_{total}$ |
| --- | --- | --- | --- | --- | --- | --- | --- |
| M155 | -1.54 | 1.13 | -0.91 | -0.24 | -1.21 | -0.34 | -1.56 |
| D156 | -1.37 | 30.72 | -28.04 | -0.20 | 1.11 | -0.01 | 1.10 |
| K159 | -7.54 | -71.99 | 74.18 | -1.21 | -6.45 | -0.11 | -6.56 |
| K160 | -1.48 | -27.25 | 27.89 | -0.23 | -0.86 | -0.21 | -1.07 |
| P163 | -0.21 | -1.94 | 1.47 | -0.01 | -0.07 | -0.63 | -0.70 |
| R169 | -0.44 | -58.46 | 53.83 | -0.23 | -5.41 | 0.12 | -5.29 |
| L193 | -0.51 | 0.96 | -0.94 | -0.11 | -0.50 | -0.10 | -0.60 |
| T196 | -0.75 | 1.13 | -0.83 | -0.18 | -0.53 | -0.10 | -0.63 |
| E200 | -1.34 | 41.66 | -38.74 | -0.27 | 1.32 | 0.00 | 1.32 |

**Table S16.** Binding free energy for tweezer/K160. Energies are in kcal/mol.

|  | complex | receptor | ligand | delta |
| --- | --- | --- | --- | --- |
| $E_{\mathrm{vdW}}$ | -1897.57(19.67) | -1847.32(19.60) | -20.23(1.15) | -30.02(3.06) |
| $E_{\mathrm{ele}}$ | -15792.31(121.38) | -16103.10(120.13) | 148.08(3.69) | 162.71(23.34) |
| $E_{\mathrm{GB}}$ | -4694.71(99.96) | -4359.51(98.11) | -181.57(2.03) | -153.63(22.55) |
| $E_{\mathrm{surf}}$ | 97.63(1.75) | 96.13(1.74) | 4.70(0.07) | -3.20(0.28) |
| $G_{\mathrm{gas}}$ | -1592.98(118.54) | -1825.34(116.88) | 99.71(6.57) | 132.65(23.80) |
| $G_{\mathrm{solv}}$ | -4597.08(99.03) | -4263.38(97.20) | -176.87(2.03) | -156.83(22.43) |
| $E_{\mathrm{gas}}+G_{\mathrm{sol}}$ | -6190.06(45.83) | -6088.72(45.27) | -77.16(6.48) | -24.18(2.86) |
| $TS_{\mathrm{total}}$ | 2625.05(7.91) | 2574.11(7.80) | 71.27(0.11) | -20.34(2.16) |
| $\Delta G_{\mathrm{bind}}^{\mathrm{cal}}$ |  |  |  | -3.84 |

# Table S17. Binding free energy decomposition per residue for tweezer/K160. Energies are in kcal/mol.

| Residues | $\Delta E_{vdW}$ | $\Delta E_{ele}$ | $\Delta E_{sol,GB}$ | $\Delta E_{sol,np}$ | $S\Delta E_{subtotal}$ | $B\Delta E_{subtotal}$ | $\Delta E_{total}$ |
| --- | --- | --- | --- | --- | --- | --- | --- |
| I157 | -1.34 | 1.85 | -1.68 | -0.16 | -1.02 | -0.30 | -1.32 |
| K159 | -1.49 | -35.76 | 36.17 | -0.28 | -0.94 | -0.41 | -1.35 |
| K160 | -8.20 | -73.89 | 74.44 | -1.42 | -7.86 | -1.21 | -9.07 |
| E161 | -1.27 | 30.18 | -28.20 | -0.14 | 0.70 | -0.13 | 0.57 |

**Table S18.** Binding free energy for tweezer/K195. Energies are in kcal/mol.

|  | complex | receptor | ligand | delta |
| --- | --- | --- | --- | --- |
| $E_{\mathrm{vdW}}$ | -1888.65(21.66) | -1832.87(21.26) | -20.78(1.08) | -35.01(3.31) |
| $E_{\mathrm{ele}}$ | -15850.79(130.08) | -16165.66(128.86) | 149.66(3.66) | 165.21(19.64) |
| $E_{\mathrm{GB}}$ | -4678.35(115.05) | -4334.13(113.97) | -182.00(1.99) | -162.22(16.42) |
| $E_{\mathrm{surf}}$ | 98.10(2.37) | 97.93(2.38) | 4.65(0.06) | -4.48(0.26) |
| $G_{\mathrm{gas}}$ | -1611.39(134.23) | -1842.25(133.25) | 100.70(6.58) | 130.16(18.77) |
| $G_{\mathrm{solv}}$ | -4580.25(113.81) | -4236.20(112.60) | -177.35(2.00) | -166.70(16.41) |
| $E_{\mathrm{gas}}+G_{\mathrm{sol}}$ | -6191.64(46.89) | -6078.44(46.58) | -76.65(6.43) | -36.54(4.47) |
| $TS_{\mathrm{total}}$ | 2623.85(9.68) | 2574.78(9.41) | 71.30(0.10) | -22.23(2.38) |
| $\Delta G_{\mathrm{bind}}^{\mathrm{cal}}$ |  |  |  | -14.31 |

# Table S19. Binding free energy decomposition per residue for tweezer/K195. Energies are in kcal/mol.

| Residues | $\Delta E_{vdW}$ | $\Delta E_{ele}$ | $\Delta E_{sol,GB}$ | $\Delta E_{sol,np}$ | $S\Delta E_{subtotal}$ | $B\Delta E_{subtotal}$ | $\Delta E_{total}$ |
| --- | --- | --- | --- | --- | --- | --- | --- |
| I191 | -1.48 | 1.19 | -1.15 | -0.20 | -1.27 | -0.37 | -1.64 |
| S192 | -1.24 | 1.72 | -1.12 | -0.16 | -0.25 | -0.54 | -0.79 |
| K195 | -7.44 | -76.18 | 77.19 | -1.14 | -6.72 | -0.85 | -7.57 |
| T196 | -1.26 | 0.87 | 0.01 | -0.15 | -0.24 | -0.30 | -0.54 |
| F198 | -1.11 | -1.20 | 1.18 | -0.07 | -0.57 | -0.63 | -1.20 |
| D199 | -1.90 | 38.19 | -35.08 | -0.33 | 0.82 | 0.06 | 0.88 |
| R224 | 0.66 | -56.6 | 48.90 | -0.19 | -7.17 | -0.05 | -7.22 |
| L227 | -1.04 | -0.73 | 0.86 | -0.10 | -0.97 | -0.05 | -1.02 |

**Table S20** Binding free energy for tweezer/K214 from docking result. Energies are in kcal/mol.

|  | complex | receptor | ligand | delta |
| --- | --- | --- | --- | --- |
| $E_{\mathrm{vdW}}$ | -1879.88(21.17) | -1827.65(20.91) | -20.28(1.22) | -31.94(3.21) |
| $E_{\mathrm{ele}}$ | -15844.18(111.44) | -16180.49(112.86) | 148.75(3.74) | 187.56(20.60) |
| $E_{\mathrm{GB}}$ | -4667.97(104.23) | -4307.44(108.35) | -182.39(2.29) | -178.13(17.83) |
| $E_{\mathrm{surf}}$ | 99.66(2.03) | 98.55(2.01) | 4.71(0.09) | -3.60(0.29) |
| $G_{\mathrm{gas}}$ | -1604.36(118.84) | -1860.38(122.36) | 100.45(6.59) | 155.58(19.65) |
| $G_{\mathrm{solv}}$ | -4568.31(103.19) | -4208.89(107.27) | -177.69(2.27) | -181.73(17.86) |
| $E_{\mathrm{gas}}+G_{\mathrm{sol}}$ | -6172.66(45.96) | -6069.27(45.30) | -77.24(6.43) | -26.15(3.46) |
| $TS_{\mathrm{total}}$ | 2628.48(7.76) | 2578.59(7.79) | 71.29(0.14) | -21.40(2.49) |
| $\Delta G_{\mathrm{bind}}^{\mathrm{cal}}$ |  |  |  | -4.75 |
